# *PCDHGC3* silencing promotes clear cell renal cell carcinoma metastasis via mTOR/HIF2α activation, lipid metabolism rewiring, and ferroptosis evasion

**DOI:** 10.1038/s41419-026-08643-y

**Published:** 2026-03-26

**Authors:** Lucía Celada, Tamara Cubiella, Jaime San-Juan-Guardado, Álvaro Suárez-Priede, Nerea Gómez-Suárez, Laura Salerno, Eduardo Murias, Marina Da Silva Torres, Joshua A. Weiner, Helena Herrada-Manchón, M. Alejandro Fernández, María-Dolores Chiara

**Affiliations:** 1 Health Research Institute of the Principado de Asturias, Oviedo, Spain; 2 Institute of Oncology of the Principado de Asturias, University of Oviedo, Oviedo, Spain; 3 Department of Medical and Surgical Sciences, University of Bologna, Bologna, Italy; 4 Radiological Service, Hospital Universitario Central de Asturias, Oviedo, Spain; 5 Department of Biology, Iowa Neuroscience Institute, University of Iowa, Iowa City, IA USA; 6 Fundación Idonial, Gijón, Spain; 7 Present Address: Fundación Idonial, Gijón, Spain

**Keywords:** Oncogenesis, Cancer metabolism

## Abstract

Clear cell renal cell carcinoma (ccRCC) remains a major clinical challenge due to its high metastatic potential and limited treatment options. Here, we identified *PCDHGC3* as a critical tumor suppressor, whose downregulation drives ccRCC aggressiveness. Through integrated molecular analyses, we demonstrated that *PCDHGC3* deficiency promoted proliferation, epithelial-to-mesenchymal transition, and metastatic dissemination in both in vitro and in vivo models. Mechanistically, *PCDHGC3* knockdown activated mTOR signaling, leading to aberrant HIF2α stabilization, a well-established oncogenic driver in ccRCC. Upstream of this cascade, *PCDHGC3* loss was associated with increased focal adhesion kinase (FAK) activation, providing a context-specific link between membrane signaling and mTOR-HIF2α pathway activation. Pharmacological inhibition of mTOR suppresses HIF2α activity and targeting either pathway partially rescues the hyperproliferative and pro-metastatic phenotype of *PCDHGC3*-deficient cells. Proteomic analysis further revealed that *PCDHGC3* loss reprograms lipid metabolism, particularly by increasing fatty acid synthesis and lipid droplet (LD) formation. We identify *PLIN2*, a HIF2α-regulated gene, as a key mediator of LD stability in *PCDHGC3*-knockdown cells. By sequestering lipids into LDs, *PLIN2* protects against ferroptosis, an iron-dependent form of cell death triggered by lipid peroxidation. Notably, *PLIN2* knockdown increases ferroptotic sensitivity, revealing LD biogenesis as a major survival mechanism in *PCDHGC3*-deficient ccRCC. Together, these findings establish a *PCDHGC3*–mTOR–HIF2α–*PLIN2* axis that underlines both metastatic behavior and ferroptosis evasion. Clinically, this suggests that combining ferroptosis inducers with mTOR or HIF2α inhibitors—and potentially targeting *PLIN2*—could provide a multifaceted therapeutic strategy against advanced ccRCC. By elucidating the tumor-suppressive role of *PCDHGC3*, this study expands our understanding of clustered *PCDH* biology and offers novel insights for ccRCC management.

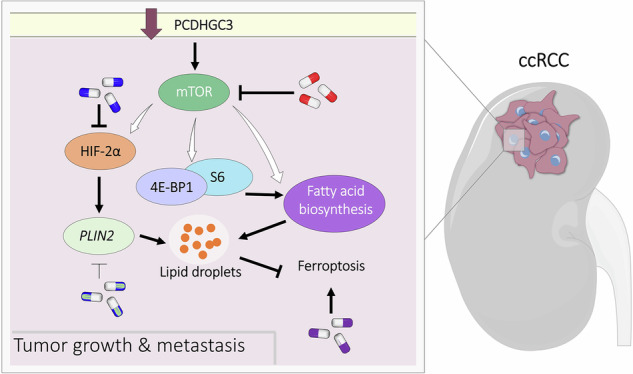

## Facts

- *PCDHGC3* acts as a tumor suppressor in ccRCC, and its loss promotes proliferation, EMT, and metastasis.
- *PCDHGC3* deficiency activates the mTOR pathway, leading to aberrant stabilization of the oncogenic factor HIF2α, and is associated with increased FAK activation upstream of this cascade.
- *PCDHGC3* loss rewires lipid metabolism by increasing fatty acid synthesis and lipid droplet formation.
- *PLIN2*, an upregulated downstream target of HIF2α, stabilizes lipid droplets and protects ccRCC cells from ferroptosis.
- Targeting *PLIN2* or combining ferroptosis inducers with mTOR/HIF2α inhibitors may offer a novel therapeutic strategy against *PCDHGC3*-deficient ccRCC.

## Introduction

Renal cell carcinoma (RCC) accounts for up to 95% of adult kidney cancers [1], with clear cell RCC (ccRCC), comprising ~75% of cases, characterized by lipid- and cholesterol-rich “clear” cells [2]. Although localized ccRCC is often managed surgically, advanced or metastatic disease remains difficult to treat and frequently becomes refractory to existing therapies [3]. Inactivation of the von Hippel-Lindau (VHL) gene, stabilization of hypoxia-inducible factors (HIFs), and alterations in the PI3K-AKT-mTOR pathway are major drivers of ccRCC [4, 5]. Targeted therapies against HIF2α, mTOR, and VEGF signaling improve survival [6–9], but resistance and relapse remain common, underscoring the need for new therapeutic targets.

Alterations in cell adhesion molecules have emerged as critical contributors to ccRCC pathogenesis. Cadherins, including classical ones like E-cadherin, regulate epithelial integrity and EMT in ccRCC [10, 11]. Less is known about protocadherins (PCDHs), particularly clustered protocadherin (cPCDH) family, primarily involved in neural development and cell-cell recognition [12–15]. The *PCDH* gene cluster, located on chromosome 5q31, encodes 53 *cPCDHs*, classified into *PCDHA*, *PCDHB*, and *PCDHG* subfamilies. These genes are increasingly recognized as tumor suppressors that maintain tissue architecture and regulate oncogenic pathways [16–20]. Notably, hypermethylation of the *cPCDH* cluster has been identified as a case of long-range epigenetic silencing, leading to widespread transcriptional repression of *cPCDH* genes in cancer [16]. Among them, *PCDHGC3* is frequently hypermethylated in colorectal cancer, neuroendocrine tumors, paraganglioma and Wilm’s tumors [16–19], yet its role in ccRCC is unknown. Given the relevance of adhesion molecules in tumor biology, we hypothesize that *PCDHGC3* loss drives ccRCC by disrupting adhesion-dependent signaling networks that regulate proliferation, invasion, and metabolic homeostasis.

Here, we investigate *PCDHGC3* regulation in ccRCC and its impact on tumor behavior. We show that *PCDHGC3* knockdown activates FAK, mTOR and HIF2α pathways, reprograms lipid metabolism, and enhances ferroptosis resistance driving aggressive tumor phenotypes. These findings uncover a novel mechanism of ccRCC progression and suggest therapeutic potential in targeting *PCDHGC3*-deficient tumors.

## Materials and methods

### Cell cultures

786-O (RRID:CVCL_1051) and RCC4 (RRID:CVCL_0498) cells, kindly provided by MJ Calzada (Universidad Autónoma, Madrid, Spain), were cultured as previously described [18]. For hypoxia experiments, cells at 70–80% confluence were exposed to 1% O_2_ for 12 h. Cell lines were authenticated by short tandem repeat profiling and routinely tested for human pathogens and mycoplasma.

### Generation of stable knockdown and cell transfections

Stable knockdown was generated using nontargeting or shRNA as previously described [18]. For *PCDHGC3* overexpression, cells were transiently transfected with a mouse-derived *PCDHGC3*-GFP vector (FL-C3-GFP) [21] using Lipofectamine LTX (Invitrogen, Thermo Fisher Scientific, Waltham, MA, USA). Empty vectors were used as controls. Transfection efficiency was quantified by flow cytometry.

### 3D cell invasion

Cell invasion was analyzed using 3D-bioprinted cancer models. Bioprinting conditions were described previously [22]. About 1 × 10^5^ cells were seeded on top of scaffolds. After spontaneous spheroid formation, microscopy imaging was conducted over a 40-h period.

### Murine models

Six-week-old female athymic nude mice (Charles River Laboratories, France) were used following the Declaration of Helsinki and University of Oviedo guidelines, approved by the Research Ethics Committee (PROAE 47/2019; PROAE 10/2020). Animals were included based on successful tumor engraftment and completion of the experimental protocol. No animals were excluded from the analysis. Investigators were not blinded to group allocation during the experiments or during outcome assessment. For tumor xenografs, 1 × 10^6^ cells were injected subcutaneously into the right flanks. Tumor size was measured biweekly and volumes calculated using 4/3π*Rr*^2^ (*R*=larger diameter, *r*=smaller diameter). Metastasis was studied using two models: 5 × 10^5^ cells injected into the tail vein or 1 × 10^6^ cells injected into the renal subcapsular space. Tumor growth and metastasis were monitored with a CT scanner (Argus CT, Sedecal, Madrid, Spain) and images reconstructed with Horos software. For drug treatments, when tumors reached approximately 100 mm^3^, mice received intraperitoneal injections of vehicle or temsirolimus (1.5 mg/kg/day) for 2 weeks (xenograft) or 3 weeks (orthotopic). Tumors and organs were visualized using the IVIS system for GFP detection.

### Proteomic analysis

Quantitative proteomic analyses were conducted at the Proteomics Service (ISPA, Oviedo, Spain). Protein extracts from 5 replicates per condition were precipitated, digested with trypsin, and analyzed by nanoLC-MS/MS using a Q-TOF ZenoTOF 7600 mass spectrometer (Sciex, Framingham, MA, USA), and an Evosep One chromatographic system (Evosep, Odense, Denmark). Statistically analysis of protein groups was performed with amica software (https://bioapps.maxperutzlabs.ac.at/app/amica). Differentially expressed proteins were identified with log2-transformed area-normalized protein levels.

### Methylation and RNAseq data analysis

Normalized RNAseq data from ccRCC samples were retrieved from TCGA and analyzed using KM plotter (https://kmplot.com/analysis/) [23]. DNA methylation profiling was performed with the Wanderer web tool (http://maplab.cat/wanderer), which provides beta values and statistical comparisons of HumanMethylation450 probes across normal and tumor samples from TCGA [24]. Bivariate associations among *PCDH* gene clusters were analyzed using AutoDiscovery software (Butler Scientifics, Barcelona, Spain), with statistical methods chosen based on data type and variable distribution, as assessed by AutoDiscovery.

### Gene/protein expression and cell growth assays

Immunohistochemistry, RNA extraction, real-time PCR (RT-qPCR), western blotting, and cell growth assays (proliferation, cell cycle and colony formation) were assessed using standard methods. Detailed procedures are provided in the Supplementary Materials.

### Lipid metabolic assay

Acetyl-CoA levels, measured with fluorometric assays, and lipid droplets density were measured as described in Supplementary Materials.

### Statistical analysis

Sample size (*n* = 8–10 animals per group) was selected a priori based on feasibility and on standard practice for tumor growth experiments in comparable models. No formal a priori power calculation was performed. Statistical analysis was performed with Graphpad Prism 10. Data are presented as mean ± SD from at least two independent experiments. Statistical significance between groups was assessed using two-tailed Student’s *t* tests. Overall survival was estimated with Kaplan–Meier analysis (log-rank test). Pearson correlation coefficients were used for correlation studies. P values < 0.05 were considered significant.

## Results

### *PCDHGC3* is uniquely regulated among *cPCDH* genes and correlates with poor prognosis in ccRCC

**Fig. 1:**
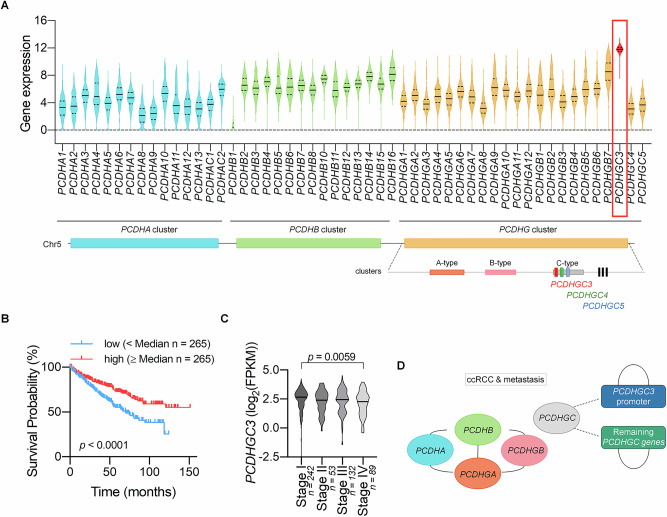
*PCDHGC3* expression in ccRCC and its correlation with disease outcome. **A** Expression levels of clustered *PCDHA*, *PCDHB* and *PCDHG* genes in ccRCC samples included in the TCGA database. An illustration in the bottom depicts the three *PCDH* gene clusters located at chromosome 5. The genomic organization of the *PCDHGC* cluster is depicted, consisting of 5′-proximal variable exons (represented as rectangles) specifically encoding *PCDHGC3* (in red), *PCDHGC4* (in green), or *PCDHGC5* (in blue), which splice to three exons (depicted as black rectangles) shared among the *PCDHG* genes. **B** Kaplan–Meier curves for ccRCC patients according to the *PCDHGC3* mRNA levels. **C** Association between *PCDHGC3* expression and disease stage. **D** Associations between CpG methylation patterns of *cPCDH* genes in metastatic ccRCCs. Analysis was performed with the Autodiscovery software.

To evaluate *cPCDH* relevance in ccRCC, we analyzed TCGA RNA-sequencing data. All available TCGA samples passing standard quality control were included in the analysis. No additional exclusion criteria were applied. Samples were analyzed without sex stratification to maximize statistical power. All *cPCDH* genes were expressed, with *PCDHGC3* showing the highest levels (Fig. 1A). Subsequent analysis revealed that reduced *PCDHGC3* expression correlated with advanced disease stage (p = 0.0059) and poorer overall survival (*p* < 0.0001) (Fig. 1B, C).

Since *cPCDH* silencing often involves promoter hypermethylation [16–20], we analyzed DNA methylation in metastatic ccRCC. *PCDHA*, *PCDHB*, and the A- and B-type *PCDHG* genes displayed coordinated hypermethylation, while C-type *PCDHG* genes, including *PCDHGC3*, did not correlate with other *cPCDH* promoters (Fig. 1D). Promoter hypermethylation was significant in A/B-type *PCDHG* genes but only subtle in *PCDHGC3* (Supplementary Fig. S1). Additionally, expression of A/B-type genes inversely correlated with promoter methylation, a relationship much weaker for *PCDHGC3* (Supplementary Fig. S1).

This distinct epigenetic and expression profile suggest *PCDHGC3* is regulated differently from other *cPCDH* genes, supporting a distinct role in ccRCC.

### *PCDHGC3* knockdown promotes ccRCC growth in vitro and in vivo

**Fig. 2:**
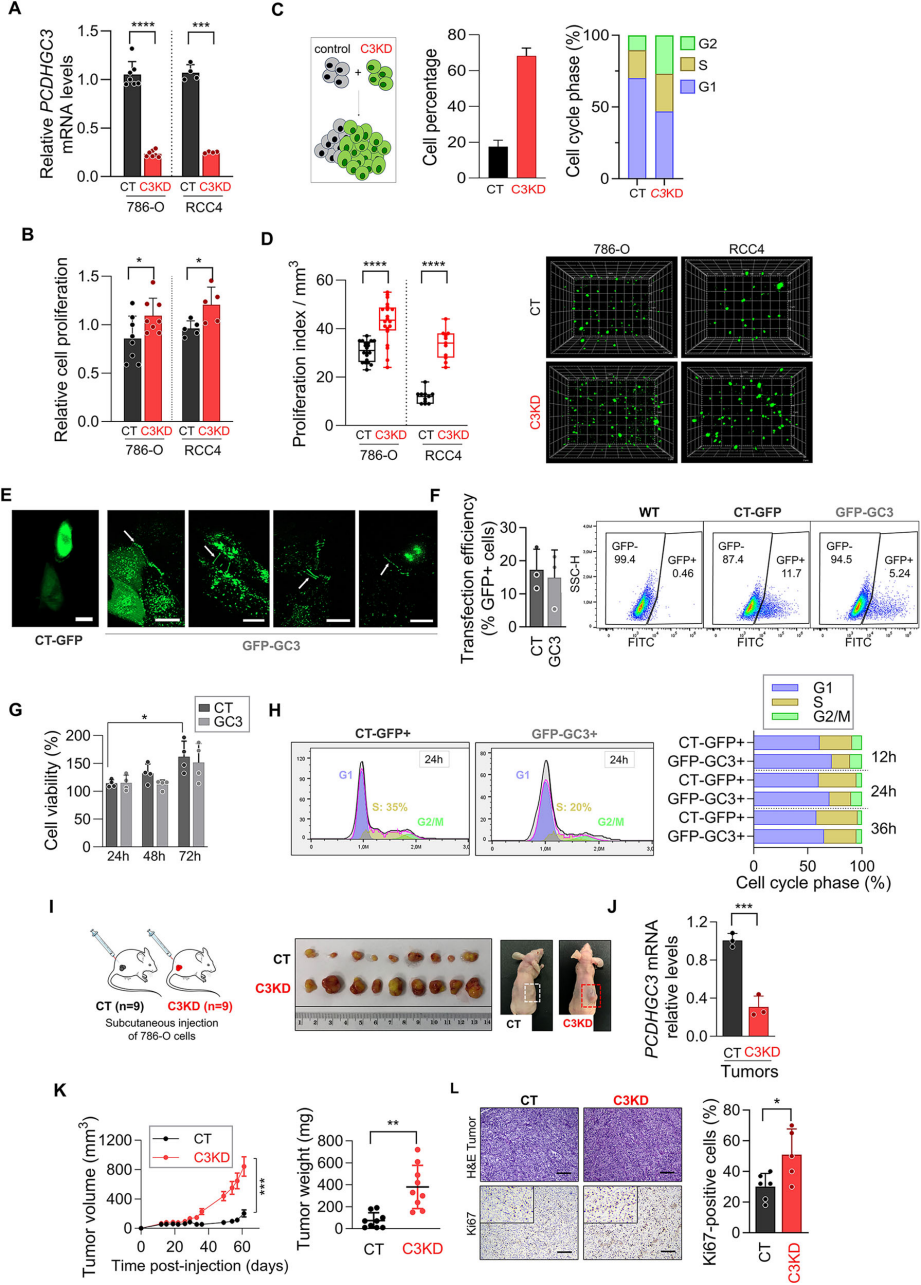
*PCDHGC3* knockdown promotes renal cancer cell proliferation in vitro and in vivo. **A** Relative *PCDHGC3* mRNA levels determined by RT-qPCR in 786-O and RCC4 cells upon shRNA-*PCDHGC3* transfection. **B** Cell proliferation of control (CT) and *PCDHGC3*-knockdown cells (C3KD) cells assessed by MTS assay over a 72-h period. **C** Schematic depiction of the experimental workflow used for the competitive proliferation assay of CT versus C3KD cells: GFP-negative CT-786-O cells transfected with nontargeting shRNA were co-cultured with GFP-positive C3KD-786-O cells at a 1:1 ratio for 4 days. The percentage of GFP-positive and negative cells was quantified by flow cytometry. Cell cycle analysis was performed separately on GFP-positive and GFP-negative populations. **D** Proliferation index of CT and C3KD cells following printing and incubation for a 14-day period. Cell counting was performed on Z-stack images (15-20 sections with an interval of 20 μm between them) at randomly chosen positions within the scaffold. Representative fluorescence XYZ projections of CT or C3KD printed cells at day 14 after printing are shown on the right. **E** Fluorescence microscopy images of 786-O cells transfected with empty vector (CT-GFP) or GFP-tagged *PCDHGC3* vector (GFP-GC3) 48-h posttransfection. White arrows point to GFP staining localized at the cell membranes and cell-to-cell contacts. **F** Percentage of GFP-expressing cells determined by flow cytometry analysis 24 hours post-transfection with CT-GFP and GFP-GC3. **G** Cell viability assessed by MTS assay in 786-O cells transfected with CT-GFP and GFP-GC3 at different time points after transfection. **H** Flow cytometry-based cell cycle analysis of the indicated cells at 12-, 24- and 36-h post-transfection. **I** Schematic representation of the mouse xenograft model and representative tumor images from CT and C3KD xenografts. **J** *PCDHGC3* mRNA levels in CT and C3KD tumor xenografts (*n* = 3 tumors per condition) **K** Volume (left) and weight (right) of tumors derived from CT or C3KD 786-O cells. Data represent mean value ± SEM (*n* = 9 tumors per group). **L** Representative images of hematoxylin & eosin (H&E) staining and Ki67 immunohistochemistry of CT and C3KD tumor xenografts. The bar plot represents the percentage of Ki67 positively stained cells. *p < 0.05, **p < 0.01, *** *p* < 0.001, **** *p* < 0.0001; Scale bars = 200 μm.

To explore *PCDHGC3*’s role in ccRCC, we used three shRNAs to knockdown its expression (C3KD) in 786-O and RCC4 cells, and nonspecific shRNA as control (CT). qPCR confirmed a reduced *PCDHGC3* transcripts in C3KD cells (Fig. 2A). Cell proliferation assays revealed a 1.27-fold and 1.25-fold increase in C3KD-786-O and C3KD-RCC4 cells, respectively (Fig. 2B). In competitive assays, in which 786-O-CT cells, not expressing GFP, and GFP-expressing C3KD-786-O cells were co-cultured, C3KD cells outcompeted CT cells over 4 days (Fig. 2C). Consistently, cell cycle analysis of GFP-positive (C3KD) and GFP-negative (CT) cells showed fewer C3KD cells in G1-phase (44.75% vs. 67.65, p < 0.01) and more in S- (29.2% vs. 18.85%, *p* = 0.02) and G2-phase (27.5% vs. 9.9%, *p* = 0.04), indicating accelerated G1/S transition (Fig. 2C). To assess whether these findings translate to a more physiologically relevant setting, we used our recently established 3D bioprinted tumor model [22] in which cells were encapsulated within hydrogels to better mimic the tumor microenvironment. Over a 15-day period, C3KD cells exhibited significantly enhanced growth compared to CT cells, with a 1.33-fold increase in 786-O and a 2.82-fold increase in RCC4 cells (Fig. 2D).

We next performed gain-of-function experiments by overexpressing GFP-tagged *PCDHGC3* in 786-O cells and using GFP-only vectors as controls (CT-GFP). Fluorescence microscopy confirmed the membrane localization of GFP-PCDHGC3 fusion protein (Fig. 2E). Although overall proliferation was unchanged, likely due to low transfection efficiency (15–20%) (Fig. 2F, G), S-phase cells were reduced by 15% upon *PCDHGC3* overexpression (Fig. 2H), supporting its role in restraining cell cycle progression.

To evaluate the impact of *PCDHGC3* knockdown in vivo, we established a xenograft model by subcutaneously injecting immunocompromised mice with C3KD or CT cells (Fig. 2I). While neither C3KD- nor CT-RCC4 cells formed tumors over 60 days, 786-O cells generated measurable tumors, with C3KD-786-O tumors exhibiting a 4.1-fold increase in volume and a 5.5-fold increase in weight compared to CT tumors (Fig. 2I-K). Immunohistochemical analysis revealed a significantly higher proportion of Ki67-positive cells in C3KD tumors (51 ± 16.73%) relative to CT tumors (30.16 ± 8.54%), indicating enhanced proliferative activity (Fig. 2L). *PCDHGC3* silencing was confirmed in xenografts via qPCR (Fig. 2J).

Overall, these results demonstrate that *PCDHGC3* silencing enhances ccRCC growth both in vitro and in vivo.

### *PCDHGC3* knockdown induces EMT, enhances survival, and promotes metastatic growth

**Fig. 3:**
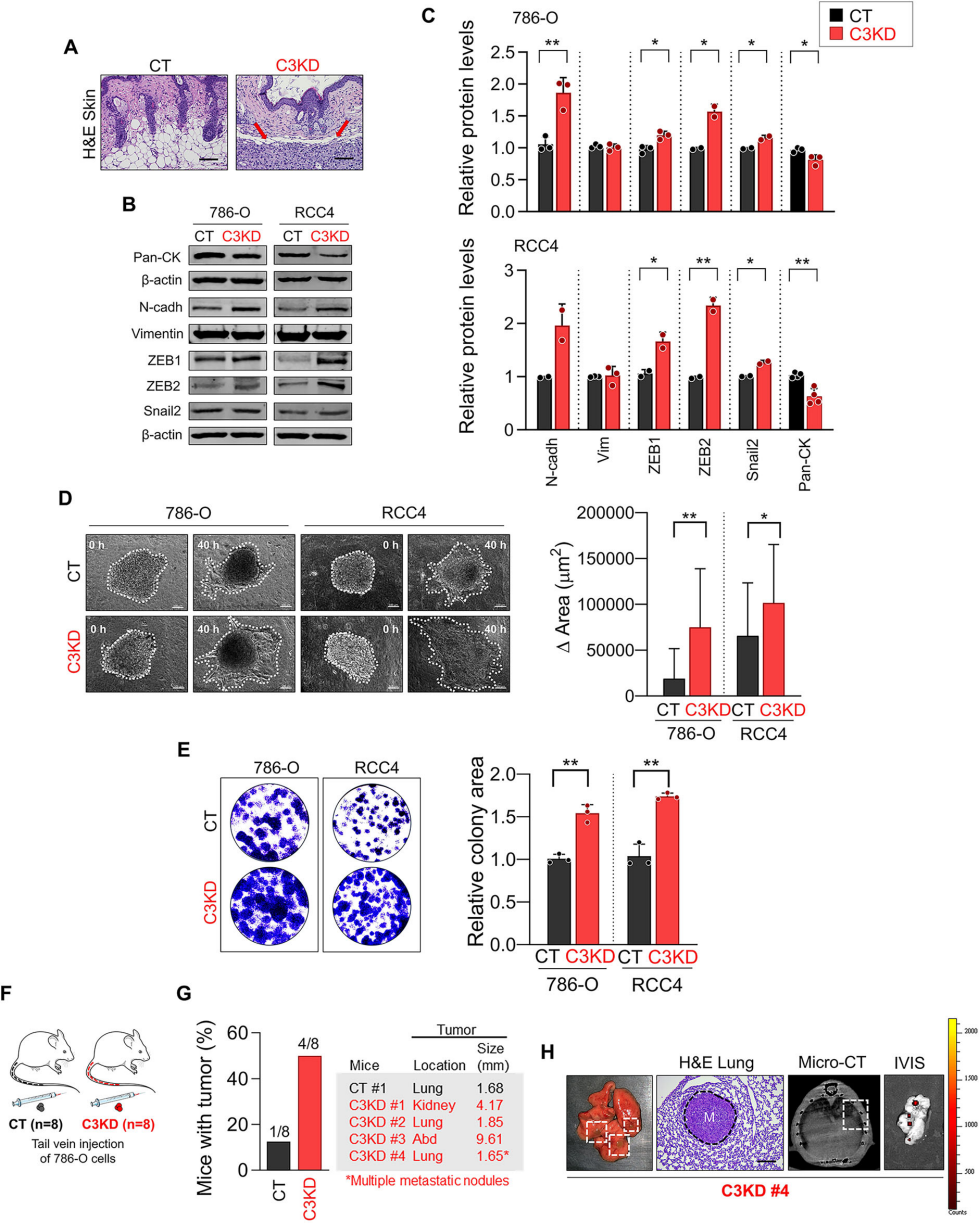
*PCDHGC3* knockdown promotes metastatic cell phenotypes. **A** Representative images of hematoxylin & eosin (H&E) staining of skin adjacent to CT and C3KD 786-O tumor xenografts. **B**, **C** Immunoblot of pan-cytokeratin (Pan-CK) or the indicated mesenchymal biomarkers in CT and C3KD cells. Expression of β-actin was used for normalization. Bar plots show the densitometric quantification of the relative protein levels. **D** Images show 786-O or RCC4 CT/C3KD cell spheroids assembled after seeding cells on top of bioink surfaces (0 h) and incubated for 40 h. The graph represents variations in spheroid invasion area over a 40-h period. For area quantification, at least eight spheroids from a minimum of seven independent experiments were used. Data are represented as mean ± SD. Scale bars: 100 μm. **E** Representative images and quantification of relative colony areas formed by 786-O and RCC4 CT and C3KD cells measured by cell colony formation assay. **F** Schematic representation of the experimental design. **G** Frequency, location, and size of tumors developed from CT and C3KD cells injected into the tail vein of mice and detected by Micro-CT scans 4 months post-inoculation. **H** Representative images of H&E staining, Micro-CT scan, and In Vivo Imaging System (IVIS) of green fluorescence in a C3KD-derived lung tumor. *p < 0.05, **p < 0.01.

To assess *PCDHGC3*’s role in metastasis, we re-examined xenograft mice for evidence of metastatic spread. While distant metastases were not detected, local invasion was observed in two C3KD tumors (Fig. 3A), but it was absent in controls suggesting increased invasiveness upon *PCDHGC3*-knockdown. Additionally, western blot analysis showed reduced cytokeratin and increased mesenchymal markers (N-cadherin, ZEB1, ZEB2, Snail2) in C3KD cells (Fig. 3B, C), indicating EMT induction. To further dissect the impact of *PCDHGC3* knockdown on metastatic progression, we evaluated its effects on three critical stages: local invasion, survival under hostile conditions, and colonization of distant organs.

In a pre-printed matrix model, cells formed spheroids that invaded the matrix over time, as previously shown [22]. Quantitative tracking of spheroid expansion over time revealed significant enhanced invasion of C3KD-786-O and C3KD-RCC4 cells (3.98-fold (*p* = 0.0023) and 1.6-fold (*p* = 0.03), respectively) compared to controls (Fig. 3D). Additionally, colony formation assays showed C3KD-RCC4 cells had 1.68-fold higher survival and growth than controls (Fig. 3E).

To evaluate in vivo colonization, we intravenously injected C3KD or CT 786-O cells into mice and monitored tumor formation over 4 months (Fig. 3F). Micro-CT revealed tumors in four of eight mice C3KD-injected mice, versus one of eight controls (Fig. 3G), confirmed by histology and IVIS imaging (Fig. 3H).

Altogether, these results show that *PCDHGC3* silencing promotes EMT, increases cell survival under stress, and enhances metastatic potential, underscoring its role as a metastasis suppressor in ccRCC.

### *PCDHGC3* loss activates mTOR and HIF2α signaling

**Fig. 4:**
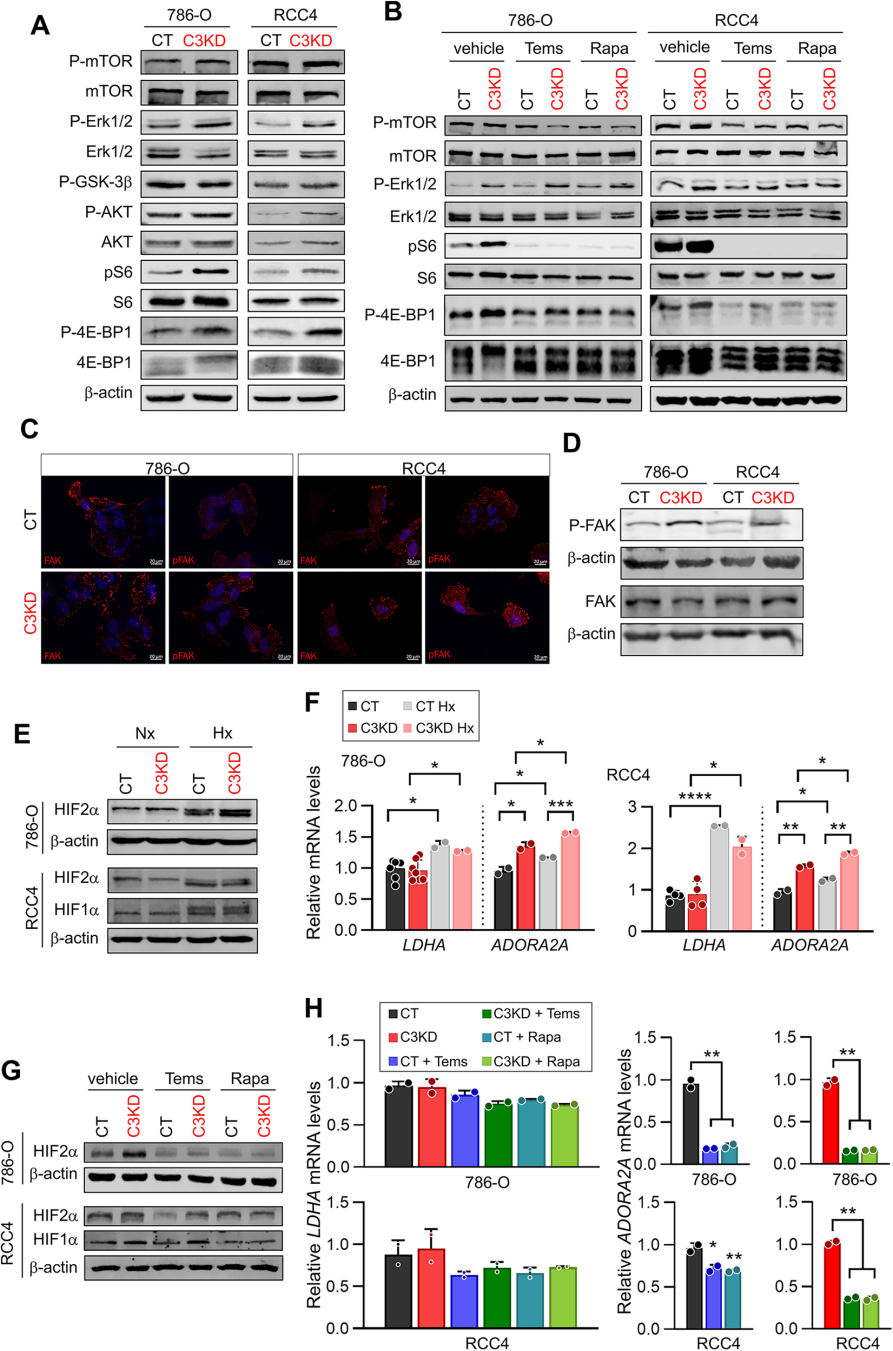
*PCDHGC3* knockdown induces activation of FAK and mTOR-HIF2α signaling. Representative immunoblots images of the indicated proteins (**A**, **B**, **D**, **E**, **G**). Cells were treated with 1 μM temsirolimus (Tems) or rapamycin (Rapa) for 24 h. **C** Immunofluorescent analysis of FAK and pFAK in the indicated cells. Hypoxia was induced by incubating cells at 1% O_2_ for 12 h. All immunoblot data are normalized to β-actin levels. **F**, **H** Relative mRNA levels of the indicated HIF target genes in 786-O and RCC4 CT and C3KD cells. Nx: normoxia, Hx: hypoxia. *p < 0.05, **p < 0.01, *** *p* < 0.001. **** *p* < 0.0001.

To uncover pathways driving the aggressive phenotype of *PCDHGC3*-deficient cells, we examined signaling cascades implicated in ccRCC, including mTOR, ERK, and Wnt [25, 26]. Western blotting showed increased phosphorylation of mTOR (pSer2448), AKT (pSer473), S6 (pSer240/244), and 4EBP1 (pThr37/46) in C3KD cells (Fig. 4A, Supplementary Fig. S2). Pharmacological inhibition of mTOR with rapamycin or temsirolimus reversed these phosphorylation events (Fig. 4B, Supplementary Fig. S2), confirming mTOR activation. C3KD cells also showed elevated pErk1/2 (pThr202/204), suggesting potential crosstalk between the Erk and mTOR pathways. In contrast, phosphorylation of GSK-3β (pSer9), a key downstream effector of the canonical Wnt signaling, remained unchanged, and immunofluorescence analysis revealed no nuclear β-catenin accumulation in C3KD cells (Supplementary Fig. S3), indicating that canonical Wnt signaling is not affected. These findings implicate mTOR and Erk -but not canonical Wnt- as downstream of *PCDHGC3* loss, though non-canonical Wnt roles remain to be explored.

Given the membrane localization of *PCDHGC3*, we next explored whether upstream adhesion-associated kinases could contribute to mTOR activation. We first examined Src signaling; however, neither total Src nor phospho-Src levels were altered upon *PCDHGC3* knockdown in either 786-O or RCC4 cells (Supplementary Fig. S4). In contrast, focal adhesion kinase (FAK) was robustly expressed in both cell lines, and *PCDHGC3*-deficient cells consistently exhibited increased FAK phosphorylation without significant changes in total FAK levels (Fig. 4C, D, Supplementary Figure S2). These data identify FAK activation as an upstream signaling event associated with *PCDHGC3* loss in ccRCC cells.

Given the well-established role of mTOR signaling in regulating HIF activity in ccRCC [27, 28], we next analyzed HIF1α and HIF2α levels (Fig. 4E, Supplementary Fig. S2). As expected, both 786-O and RCC4 cells exhibited constitutive HIF accumulation due to loss of functional pVHL [29]. 786-O cells exclusively express HIF2α, whereas RCC4 cells express both HIF1α and HIF2α. Exposure to hypoxia further increased the levels of both isoforms in RCC4 cells and of HIF2α in 786-O cells. Notably, HIF2α was significantly upregulated in C3KD-786-O cells, with milder effects in RCC4. In contrast, HIF1α levels were unchanged, and *LDHA*, a classical HIF1α target, remained stable. In contrast, *ADORA2A*, a HIF2α target, was upregulated in both C3KD lines (Fig. 4F). mTOR inhibitors reduced HIF2α stabilization and selectively downregulated *ADORA2A* without affecting HIF1α or *LDHA* (Fig. 4G, H), indicating that mTOR contributes to HIF2α upregulation.

We next validated these findings in the tumor xenografts, where *PCDHGC3* knockdown consistently activated mTOR and HIF2α pathways as evidenced by signaling protein analysis (Supplementary Fig. S5). Immunohistochemistry confirmed higher nuclear HIF2α in C3KD tumors (Supplementary Fig. S5). However, unlike in vitro, ERK activation was not observed, suggesting context-dependent signaling.

### mTOR and HIF2α pathways drive proliferation in *PCDHGC3*-deficient cells

**Fig. 5:**
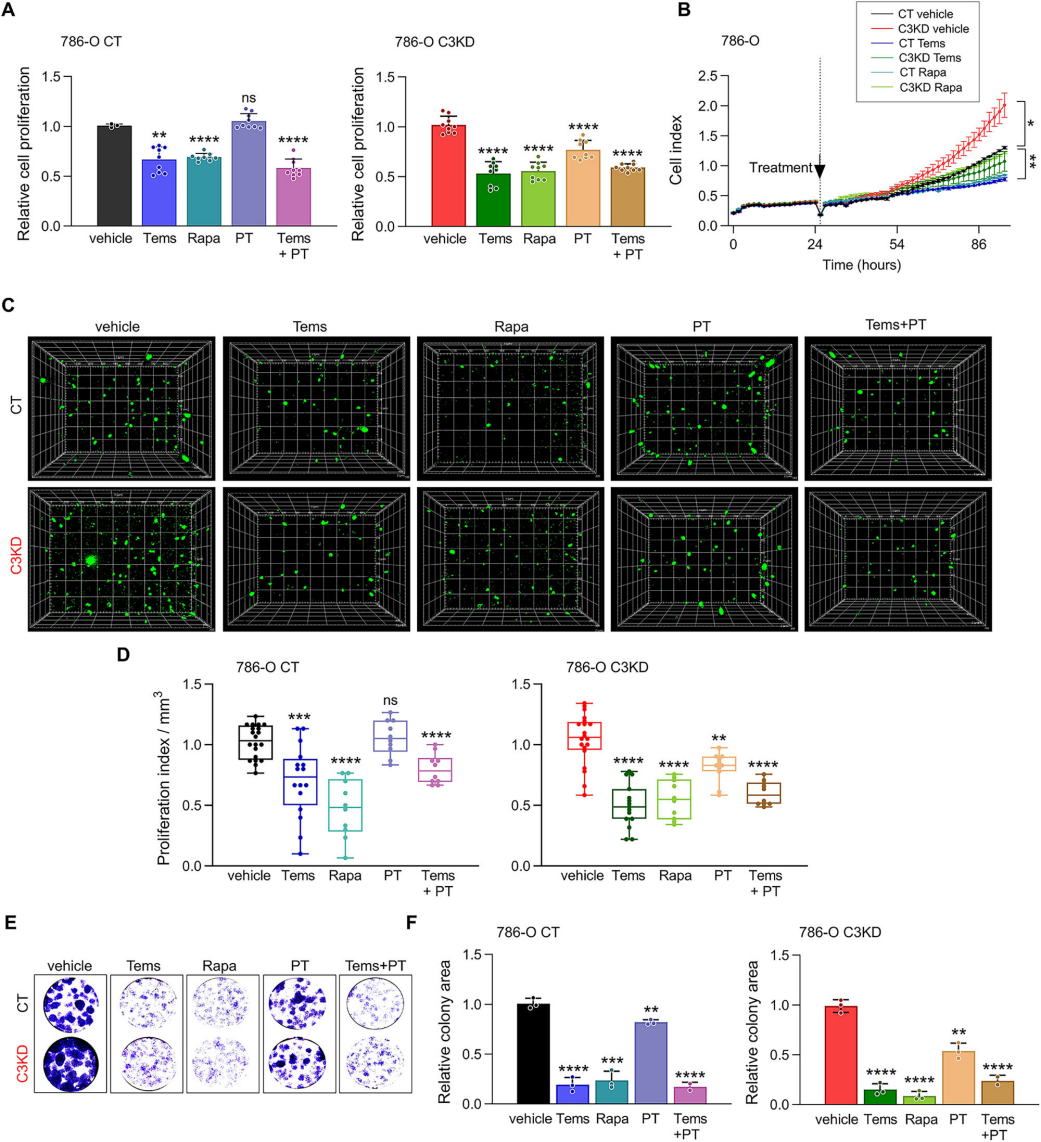
mTOR and HIF2α inhibitors suppress high proliferative rate induced by *PCDHGC3* deficiency in ccRCC cells. **A** MTS cell proliferation of the indicated cells 72 h after treatment with 1 μM temsirolimus (Tems), 1 μM rapamycin (Rapa) or 50 μM PT2385 (PT). **B** Real-time analysis of cell proliferation using the iCELLigence system in 786-O CT or C3KD cells treated or not with temsirolimus or rapamycin (1 μM). **C** Representative fluorescence XYZ projections of 786-O CT or C3KD printed cells treated with the indicated drugs. **D** Quantification of bioprinted cells after treatment with different drugs. Cell counting was performed on Z-stack images (15-20 sections with an interval of 20 μm between them) at randomly chosen positions within the scaffold. **E**, **F** Representative images and quantification of relative colony areas formed by CT and C3KD 786-O cells measured by colony formation assay. DMSO was used as a vehicle in all assays. Data show mean value ± SD (*n* ≥ 3 independent experiments). *p < 0.05, **p < 0.01, *** *p* < 0.001, **** *p* < 0.0001.

To assess whether mTOR and HIF2α activation underlies the hyperproliferation of C3KD cells, we treated 786-O cells with rapamycin, temsirolimus, or the HIF2α inhibitor PT2385. Cell growth was measured after 3 days using MTS assays (Fig. 5A) and in real-time via iCelligence (Fig. 5B). Both mTOR inhibitors reduced proliferation in C3KD and CT cells, with C3KD cells showing a 1.22-1.38-fold greater reduction (p < 0.04) (Fig. 5A, B**;** Supplementary Fig. S6). PT2385 selectively impaired C3KD proliferation by 1.32-fold (p < 0.0001), with no effect on CT cells. Combined treatment showed no additive effect, suggesting convergence of mTOR and HIF2α pathways in regulating proliferation (Fig. 5A). Similar results were observed in 3D bioprinted tumor models and colony formation assays (Fig. 5C–F**;** Supplementary Fig. S6).

### Temsirolimus shows therapeutic efficacy in *PCDHGC3*-deficient tumors

**Fig. 6:**
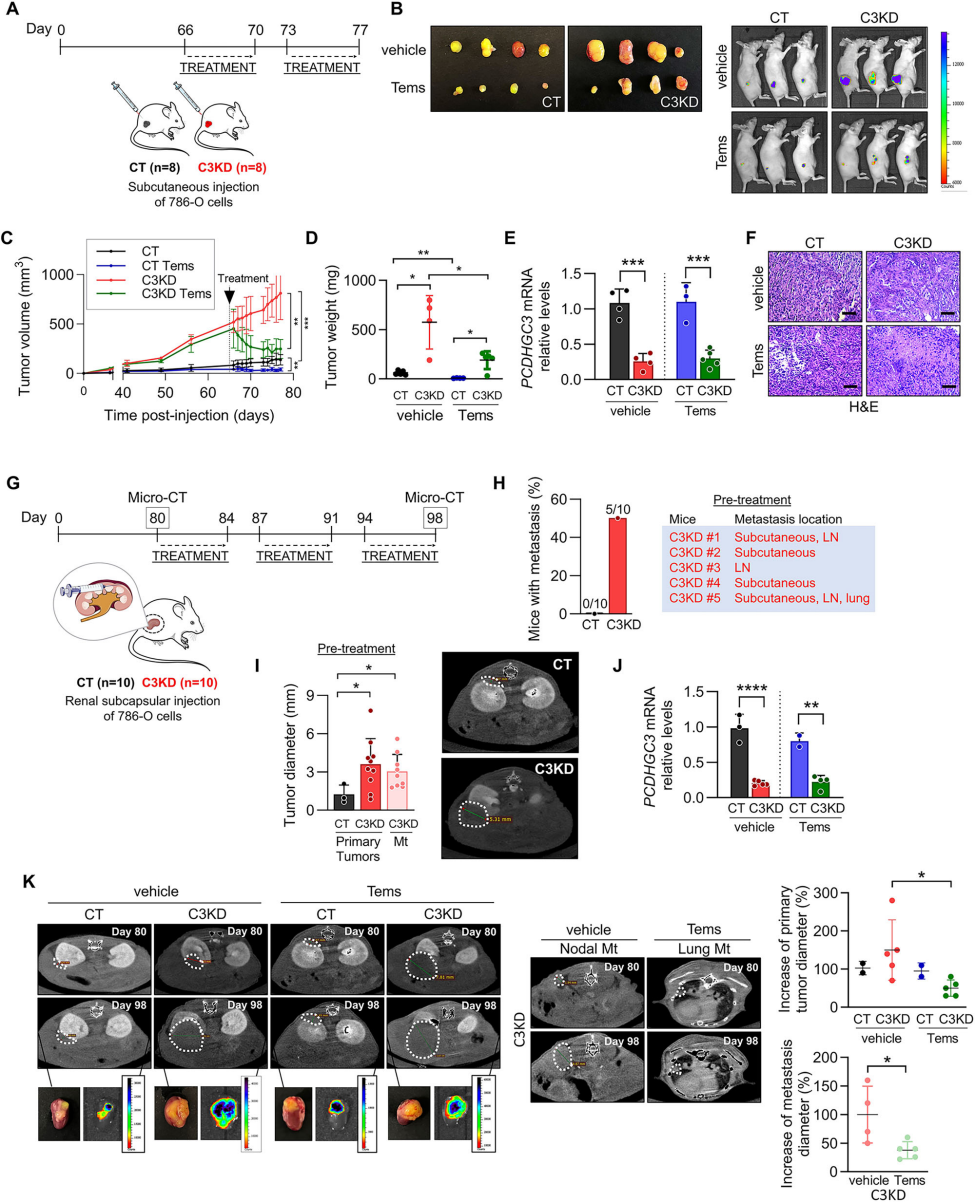
mTOR inhibition reduces the enhanced tumor growth and prevents metastasis development induced by *PCDHGC3* silencing in vivo. **A**–**F** Tumor xenograft model. **A** Schematic overview of the experimental procedure in the mouse xenograft model. Vehicle (DMSO) or temsirolimus (Tems) were administered intraperitoneally 66 days after cell inoculation in athymic nude mice at a dosage of 1.5 mg/kg/day, 5 days/week for 2 weeks. **B** Representative images of tumor xenografts and IVIS detection of green fluorescence in the whole animals after treatments. Tumor volume (**C**) and weight (**D**) of the indicated tumor xenografts under the specified treatments. Data represent mean value ± SEM. **E** Relative *PCDHGC3* mRNA levels measured in tumor xenografts after treatments. **F** Representative image of H&E staining of the indicated tumors xenografts. **G**-**K** Orthotopic model. **G** Flowchart of the experimental design. Vehicle (DMSO) or temsirolimus (1.5 mg/kg/day) treatments were administered intraperitoneally 5 days a week for 3 weeks. **H** Pretreatment analysis by Micro-CT showing the percentage of mice with metastasis and the metastasis location. LN: lymph nodes. **I** Bar chart represents the mean diameter (mm) ± SD of primary tumor and metastases (Mt) measured 80 days after cell inoculation by analysis of Micro-CT. Representative images of Micro-CT scans of the indicated tumors before treatments are shown. **J** *PCDHGC3* mRNA levels in the indicated tumors after treatments. **K** Representative Micro-CT scans of mice before (Day 80) and after (Day 98) treatment showing primary tumors and metastases. Response to treatments is shown as the variation of tumor diameter between days 80 and 98 in mice treated with vehicle or temsirolimus. Scale bars = 200 μm. *p < 0.05, **p < 0.01, *** *p* < 0.001. **** *p* < 0.0001.

To assess the translational potential of mTOR inhibition, we tested temsirolimus in two mouse models. In subcutaneous xenografts, 786-O tumors were treated after 66 days using two 5-days cycles (Fig. 6A). Although temsirolimus reduced tumor size and weight (Fig. 6B–D), there was no significant difference in drug sensitivity between C3KD tumors and controls (tumor reduction of 1 to 26-fold in C3KD xenografts vs. 5 to 11-fold in control xenografts, *p* = 0.990). The reduced levels of *PCDHGC3* mRNA were confirmed in the xenografts (Fig. 6E). The only distinct feature in treated C3KD tumors was increased necrosis and fibrosis (Fig. 6F). Thus, *PCDHGC3*-deficient tumors did not exhibit greater sensitivity to temsirolimus in this model. This likely reflects the limited physiological relevance of the microenvironment in subcutaneous tumors.

We next used an orthotopic model by injecting cells under the renal capsule (Fig. 6G). By day 80, metastases developed in 5 out of 10 mice with C3KD tumors, whereas no metastases were detected in control mice (Fig. 6H). C3KD tumors were also significantly larger (2.93-fold, *p* = 0.01) (Fig. 6I). Temsirolimus treatment reduced primary tumor size by 67% and metastatic burden by 62% in C3KD-bearing mice, while control tumors were unresponsive (Fig. 6K). *PCDHGC3* knockdown and altered p-S6, p-4E-BP1, and HIF2α levels were confirmed in treated tumors and metastases (Fig. 6J; Supplementary Fig. S7).

These results indicate that *PCDHGC3*-deficient tumors are more responsive to mTOR inhibition in an orthotopic, metastatic setting.

### *PCDHGC3* deficiency induces lipid metabolism reprograming

**Fig. 7:**
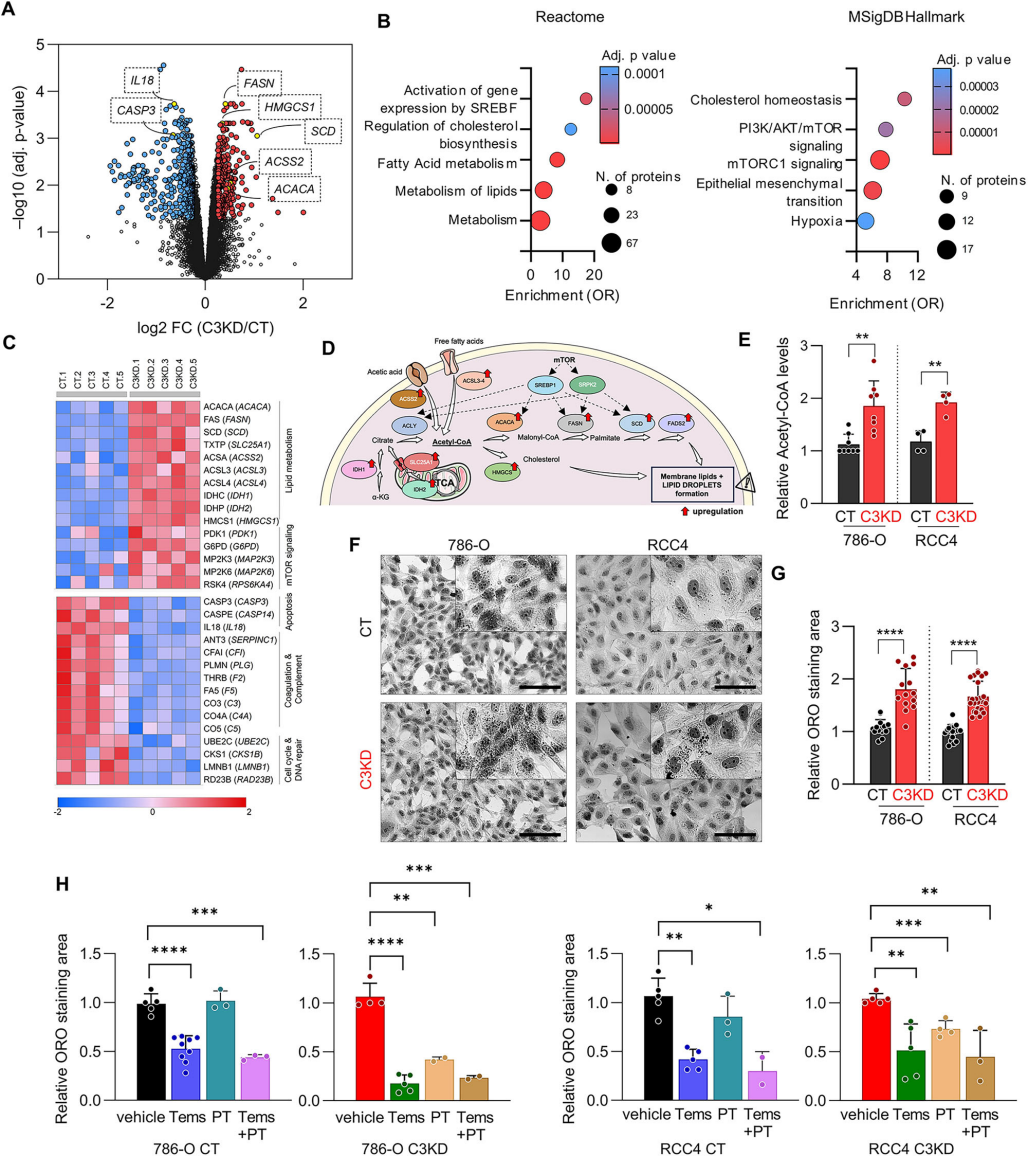
Lipid metabolism reprograming following *PCDHGC3* knockdown. **A** Volcano plot representation of proteins identified by proteomic analysis of C3KD versus CT 786-O cells. Proteins significantly up- or down-expressed (adjusted *p* value < 0.01 and > 1.2-fold-cutoff) are highlighted by red or blue circles, respectively. **B** Enrichment analysis of up-regulated proteins using Reactome and Molecular Signature (MSigDB) Hallmarks databases. **C** Heatmap visualization of the most significantly differentiated proteins. **D** Schematic representation of the pathways involved in lipid and cholesterol synthesis. Red arrows indicate proteins upregulated in 786-O C3KD cells compared to CT cells. **E** Relative intracellular acetyl-CoA levels in CT and C3KD cells. **F**, **G** Representative images of phase-contrast microscopy of the indicated cells stained with Oil Red O (ORO) 3 days after reaching confluence. Bar charts show quantification of ORO area staining. **H** Quantification of ORO staining in the indicated cells. Temsirolimus (1 μM) and PT2385 (50 μM) treatments were performed for 72 h once cells reached confluence. Scale bars = 100 μm. *p < 0.05, **p < 0.01, ****p < 0.0001.

To explore broader signaling changes in C3KD cells, we performed quantitative proteomics in 786-O cells. Of 7,350 proteins identified, 292 were upregulated and 458 downregulated in C3KD *versus* CT (fold change ≥1.2, adjusted *p* < 0.01) (Fig. 7A; Supplementary Table S1). Pathway analysis revealed enrichment of mTORC1, HIF, and EMT signatures, along with strong upregulation of lipid metabolism-related proteins (Fig. 7B–D). Notably, key enzymes in fatty acid (FA) synthesis -SLC25A1, ACACA, FASN, SDC, and FADS2- and acetyl-CoA biosynthesis -ACSS2, ACSL3- were significantly elevated (Fig. 7D; Supplementary Table S2). However, mitochondrial function remained unchanged based on the Cell Mito Stress Seahorse assay (Supplementary Fig. S8). These results suggest that *PCDHGC3* loss promotes lipid biosynthesis, particularly via acetyl-CoA. Consistently, C3KD cells exhibited elevated acetyl-CoA levels (1.7-fold, *p* < 0.002) and lipid droplet (LD) accumulation (1.7-fold, *p* < 0.0001) as compared to CTs (Fig. 7E–G).

To further investigate the regulation of LD formation in C3KD cells, we treated cells with temsirolimus or PT2385. LD density dropped significantly, especially in C3KD-786-O cells (6-fold versus 1.87-fold in CTs) (Fig. 7H**;** Supplementary Fig. S9), indicating mTOR-dependent lipid accumulation. HIF2α inhibition with PT2385 also reduced LDs in C3KD cells, more strongly in 786-O (HIF2α-only) than RCC4, while CT cells were unaffected. Combined treatment showed no additive effect, indicating that *PCDHGC3*-deficient cells exhibit a unique dependence on HIF2α-driven lipid metabolism.

### *PLIN2*-mediated lipid droplet accumulation confers ferroptosis resistance in *PCDHGC3*-deficient ccRCC

**Fig. 8:**
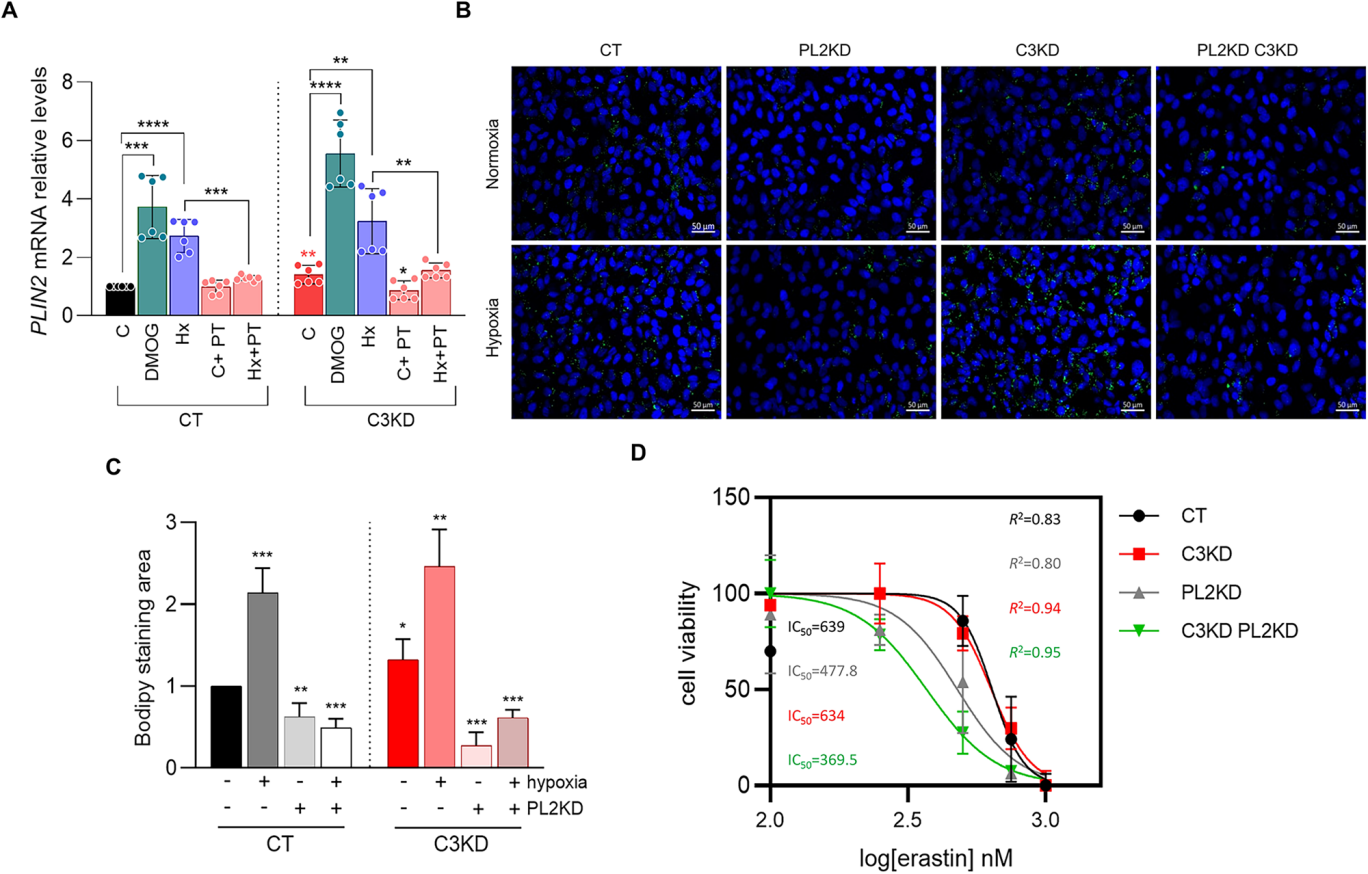
*PLIN2* knockdown sensitizes *PCDHGC3*-deficient cells to ferroptosis. **A** *PLIN2* mRNA levels in CT and C3KD cells cultured under normoxia (C) or hypoxia (Hx, 1% O_2_) with or without the HIF2α inhibitor, PT2385 (50 μM; C + PT, Hx+PT). To further assess HIF-dependent regulation of *PLIN2*, cells were treated with the prolyl hydroxylase inhibitor DMOG (1 mM, 24 h) to stabilize HIFα. **B** Representative images of BODIPY 493/503 staining in the indicated cells. *PLIN2* was knocked down in control (CT) and *PCDHGC3*-knockdown (C3KD) cells using *PLIN2*-targeting shRNA, generating PL2KD and PL2KD C3KD cells, respectively. **C** Bar chart illustrating the relative quantification of the BODIPY-stained area, reflecting lipid droplet accumulation across conditions. **D** CT, C3KD, PL2KD, and double-knockdown (PL2KD C3KD) cells were treated with erastin, a ferroptosis inducer, and assessed for ferroptotic cell death. Scale bars = 50 μm. *p < 0.05, **p < 0.01, **** *p* < 0.0001. Statistical significance between C3KD and CT conditions is indicated by red asterisks.

HIF signaling is a well-established regulator of lipid metabolism, in part through the transcriptional upregulation of *PLIN2*, which encodes perilipin-2, a key protein involved in LD formation and stabilization. Although perilipin-2 was not detected in our proteomic analysis, we confirmed *PLIN2* overexpression in C3KD cells compared to CTs (Fig. 8A). Consistent with previous reports [30], *PLIN2* expression was induced by HIF2α activation under hypoxia and DMOG-induced pseudohypoxia, an effect that was abrogated by PT2385.

To directly assess *PLIN2*’s role in LDs formation, we quantified LD density by BODIPY 493/503 staining in cells treated with *PLIN2*-targeting shRNA. LD accumulation was significantly reduced in C3KD cells but only mildly affected in CT cells (Fig. 8B, C). Under hypoxia, *PLIN2* depletion abolished LD accumulation in both CT and C3KD cells, confirming its essential role in hypoxia-driven lipid storage.

Based on these findings, we hypothesized that *PLIN2* upregulation protects C3KD cells from ferroptosis by sequestering excess FAs into LDs, limiting lipid peroxidation. To test this, we generated *PLIN2* knockdown (PL2KD) and double knockdown (PL2KD C3KD) cells using *PLIN2*-targeting shRNA. Cells were then treated with erastin, a ferroptosis inducer (Fig. 8D). PL2KD cells showed increased ferroptosis sensitivity, supporting *PLIN2*’s protective role. Notably, while C3KD and CT cells responded similarly to erastin, double-knockdown cells showed the highest susceptibility to ferroptosis, indicating that *PLIN2* upregulation in C3KD cells acts as a critical defense mechanism by preventing the accumulation of free peroxidizable lipids. These results highlight *PLIN2* as a key mediator of metabolic adaptation and survival in *PCDHGC3*-deficient ccRCC and reveal a therapeutic vulnerability.

## Discussion

This study uncovers a novel mechanism driving metastatic ccRCC, showing for the first time that *PCDHGC3* downregulation promotes tumor growth, invasion, and metastasis. While clustered protocadherins (*cPCDHs*) have been implicated in various cancers [16–20], our findings establish the first direct link between *PCDHGC3* loss and metastatic progression in ccRCC.

Using in vitro and in vivo models, along with proteomic profiling, we demonstrate that *PCDHGC3* deficiency reprograms tumor cells toward a more aggressive phenotype, marked by enhanced proliferation, cell-cycle dysregulation, EMT, and increased metastatic potential. Although *PCDHGC3* silencing has been reported in colon cancer [16], aggressive paraganglioma/pheochromocytoma [18], and neuroendocrine carcinoma [19], its direct involvement in metastasis was previously unproven. Our data now provide clear mechanistic evidence that *PCDHGC3* acts as a metastasis suppressor, and that its loss may be a critical event in ccRCC progression.

Notably, *PCDHGC3* appears uniquely regulated among *cPCDHs*, suggesting it functions independently rather than within a broader protocadherin network. This specificity mirrors findings from mouse models, where individual C-type protocadherins play distinct, non-redundant roles in neural development. For example, α-*Pcdh*-C2 is essential for serotonin-producing neuron projections across multiple brain regions [31], γ-*Pcdh*-C3 regulates dendritic arborization and synapse formation [32, 33], and γ-*Pcdh*-C4 is the only clustered Pcdh protein strictly required for neuronal survival [34, 35]. Similarly, our findings suggest that *PCDHGC3* has a specialized role outside the nervous system, acting as a key regulator of tumor progression and metastatic competence in ccRCC.

Mechanistically, *cPCDHs* have been linked to multiple signaling pathways, including Wnt, mTOR, and adhesion-associated signaling networks [16, 36–38]. Our findings identify mTOR and HIF2α, both commonly activated in ccRCC, as key downstream effectors of *PCDHGC3* loss. While mTOR promotes cell growth and survival, HIF2α regulates angiogenesis, metabolism, and tumor progression [4, 39, 40]. We demonstrate that pharmacological inhibition of either pathway reverses the proliferative and metastatic phenotypes of *PCDHGC3*-deficient cells; however, their combined inhibition offers no additive benefit, suggesting convergence downstream of *PCDHGC3* [41].

While these data establish a functional link between *PCDHGC3* loss and activation of oncogenic mTOR-HIF2α signaling, the molecular events connecting this membrane protein to intracellular signaling cascades have remained poorly defined in cancer. Previous studies in neuronal systems have demonstrated that γ-Pcdh signal through their constant cytoplasmic domain to activate focal adhesion FAK, engaging downstream PKC–MARCKS signaling pathways that regulate cell adhesion and survival [36, 37, 42]. Consistent with this established role of γ-Pcdh in FAK-dependent signaling, we find that loss of *PCDHGC3* in ccRCC cells is associated with increased phosphorylation of FAK at Y397, the major site of FAK activation. In contrast, Axin1, a scaffold protein reported to interact with γ-Pcdh-C3 in neurons and implicated in Wnt and mTOR signaling [32, 38, 43], was expressed at very low levels in our ccRCC models, and no interaction with PCDHGC3 was detected, suggesting that distinct adapter mechanisms may couple PCDHGC3 to intracellular signaling pathways in epithelial tumor cells.

Together, these signaling alterations help explain the metabolic phenotype associated with PCDHGC3 deficiency, including enhanced fatty acid synthesis, increased acetyl-CoA levels, and lipid droplet accumulation. These results align with the lipid-rich phenotype of ccRCC [2, 44], where elevated FA metabolism is associated with poor prognosis [45–51]. Notably, HIF2α emerged as the primary driver of LD accumulation in C3KD cells. While mTOR inhibition affected both CT and C3KD cells, the HIF2α inhibitor PT2385 selectively reduced LDs in C3KD cells, suggesting that HIF2α-mediated lipid storage is a tumor-specific adaptation to PCDHGC3 loss. Thus, HIF2α is essential for metabolic reprogramming in this context, while mTOR has broader effects on lipid metabolism.

We identify *PLIN2* as a crucial mediator of LD accumulation and a downstream effector of mTOR-HIF2α signaling. Our findings reveal that *PLIN2* depletion sensitized *PCDHGC3*-deficient cells to ferroptosis, indicating that perilipin-2-driven LD formation protects against lipid peroxidation by sequestering free FAs. This adaptive mechanism supports tumor survival under stress and reveals a metabolic vulnerability.

Targeting *PLIN2* or disrupting LD formation could render *PCDHGC3*-deficient tumors more susceptible to ferroptosis-inducing therapies, offering a promising treatment strategy. A dual-targeting approach combining ferroptosis inducers (e.g., RSL3) with perilipin-2 inhibition could potentiate cell death and limit metabolic adaptation. Additionally, the upregulation of FA metabolism in *PCDHGC3*-knockdown cells suggests that targeting lipogenic enzymes such as FASN, ACC, or ACLY may further impair tumor progression and sensitize cells to ferroptosis.

HIF2α inhibition also represents a promising therapeutic strategy. Given that *PLIN2* is regulated downstream of mTOR–HIF2α, targeting HIF2α could disrupt lipid homeostasis in PCDHGC3-deficient tumors. Belzutifan (MK-6482), a selective HIF2α inhibitor now FDA-approved for VHL-associated ccRCC [39, 52–54], may therefore represent a rational therapeutic option. Combining belzutifan with ferroptosis inducers or FA metabolism inhibitors may enhance tumor vulnerability to oxidative stress, supporting its use as a precision therapy in *PCDHGC3*-deficient ccRCC.

In summary, this study is the first to establish *PCDHGC3* as a metastasis suppressor in ccRCC. We demonstrate that its downregulation orchestrates a complex reprogramming of mTOR-HIF2α signaling and FA metabolism, culminating in enhanced tumor growth, invasion, and lipid metabolism rewiring. By delineating the *PCDHGC3*–mTOR–HIF2α–*PLIN2* axis, we uncover multiple potential intervention points -from targeted kinase and HIF inhibitors to ferroptosis inducers and FA synthesis blockers- that may inform personalized therapy for aggressive ccRCC. We propose that *PCDHGC3*-deficient tumors develop a metabolic adaptation that shields them from ferroptosis via *PLIN2*-mediated LD accumulation. However, this adaptation also creates an exploitable vulnerability, where *PLIN2* depletion sensitizes these tumors to ferroptotic cell death. These findings underscore the importance of expanding our understanding of *cPCDH* family members beyond their canonical roles in neurobiology and highlight *PCDHGC3* as a promising biomarker and therapeutic target in advanced kidney cancer.

## Supplementary information

Supplementary Materials

Supplementary Figure S1

Supplementary Figure S2

Supplementary Figure S3

Supplementary Figure S4

Supplementary Figure S5

Supplementary Figure S6

Supplementary Figure S7

Supplementary Figure S8

Supplementary Figure S9

Table S1

Table S2

Uncropped western blots

## Data Availability

The datasets generated and/or analyzed during the current study are available from the corresponding author on reasonable request.
