# Supplementary material for: PCDHGC3 silencing promotes clear cell renal cell carcinoma metastasis via mTOR/HIF2α activation, lipid metabolism rewiring, and ferroptosis evasion
Source: Cell Death Dis. 2026 Mar 26;17(1):409. doi: 10.1038/s41419-026-08643-y (PMC13144475; doi:10.1038/s41419-026-08643-y)
Supplement: Supplementary file 1 — Supplementary Materials [file 41419_2026_8643_MOESM1_ESM.docx]

***PCDHGC3* silencing promotes clear cell renal cell carcinoma metastasis via mTOR/HIF2α activation, lipid metabolism rewiring, and ferroptosis evasion**

Lucía Celada, Tamara Cubiella, Jaime San-Juan-Guardado, Álvaro Suárez-Priede, Laura Salerno, Eduardo Murias, Marina Da Silva Torres, Joshua A. Weiner, Helena Herrada-Manchón, M. Alejando Fernández, María-Dolores Chiara

**Supplementary Materials**

**Chemicals and reagents**

Temsirolimus (S1044) and rapamycin (S1039), were purchased from Selleckchem (Houston, TX, USA), and PT2385 and erastin (HY-15763) from MedChemExpress (New Jersey, USA). Stocks were prepared at 10 mM in sterile DMSO. GIPZ lentiviral human *PCDHGC3* and *PLIN2* shRNA plasmids and non-silencing control were purchased from Horizon Discovery (Cambridge, UK).

**Cell proliferation and cell cycle assays**

Cell growth and viability were assessed using the MTS CellTiter 96® AQueous One Solution Cell Proliferation Assay (Promega, Madison, Wisconsin, US) after 72 hours of treatments, following the manufacturer’s protocol. Real-time cell proliferation was monitored with the xCELLigence RTCA eSight Real-Time Cell Analyzer (RTCA) (RRID:SCR_019571, Agilent Technologies, Santa Clara, CA, USA), where 3 x 10^3^ cells were seeded on biosensor plates. Impedance, which correlates with cell proliferation and adhesion, was measured every two hours for 96 hours. Drugs were added 26 hours after seeding. For 3D models, cells were embedded in bioinks and printed as scaffolds [24]. Drug treatments were applied 24 hours post-printing, and cell growth was tracked via microscopy and cell counting on Z-stack images before and after 14 days of treatment. For competitive proliferation assays, GFP-expressing C3KD cells were mixed at a 1:1 ratio with control cells, which lacked GFP expression but had been stably transfected with a control shRNA. After 4 days of co-culture, the percentage of GFP-positive/negative cells was quantified by flow cytometry. Cell cycle analysis was performed separately on GFP-positive and GFP-negative populations. For cell cycle analysis, cells were fixed and stained with 1 μg/mL DAPI before flow cytometry. Data were analyzed using FlowJo software.

**Colony formation assays**

Cells were seeded at low density (150 cells) in twelve-well plates. After 24 hours, they were treated with drugs and cultured for 7 days. Cells were fixed with ice-cold methanol, stained with 0.5% crystal violet, and colony coverage was quantified using an ImageJ plugin.

**Immunohistochemistry and immunofluorescence**

Antigen retrieval was performed using EnVision™ FLEX target retrieval solution on a Dako PT link platform (Dako Denmark A/S, Glostrup, Denmark). Staining was done with Dako EnVision™ Flex detection system. Antibodies were used as follows: anti-HIF2α (Abcam Cat# ab199, RRID:AB_302739, Cambridge, UK) at 1:50 dilution for 30 min and anti-Ki67 (Agilent Cat# M7240, RRID:AB_2142367, Agilent Technologies, Santa Clara, CA, USA) at 1:100 dilution for 20 min. Fluorescence microscopy was performed with a Zeiss Axio Observer (RRID:SCR_021351) microscope (Carl Zeiss, Germany). For immunofluorescence, cells were plated in 96-well plates with square bottoms (µ-Plate 96 well square, Ibidi, GmbH, Gräfelfing, Germany) and fixed with 100 µL of 4% paraformaldehyde for 15 minutes. Primary antibodies were incubated overnight at 4°C with the primary antibody diluted in 1X PBS containing 1% BSA and 0.3% Triton X-100. The primary antibodies used were: mouse anti-FAK, clone 4.47 (Millipore Cat# 05-537, RRID:AB_2173817) at 1:100 dilution and mouse anti-human FAK (pY397) (BD Biosciences Cat# 611722, RRID:AB_399198) at 1:100 dilution, and mouse anti-human β-catenin (BD Biosciences Cat# 610153, RRID:AB_397554) at 1:100 dilution. For secondary antibody staining, goat anti-mouse IgG (H+L), highly cross-adsorbed secondary antibody, Alexa Fluor™ 488 (Thermo Fisher Scientific Cat# A-11029, RRID:AB_2534088) was used at 1:500 diultion. To stain the DNA, DAPI solution (Ibidi, GmbH) was added to the cells. Finally, imaging was performed using a Zeiss AxioObserver Z1 microscope (RRID:SCR_021351, Carl Zeiss, Germany).

**RNA extraction and real-time PCR (RT-qPCR)**

Total RNA was isolated from cells with mirVanaTM miRNA Isolation Kit (Invitrogen, Thermo Fisher Scientific, Waltham, MA, USA). cDNA was synthesized from 100 ng of RNA with the Maxima First Strand cDNA synthesis kit for RT-qPCR (Thermo Fisher Scientific). Gene expression was analyzed using TaqMan PCR Master Mix (Applied Biosystems, Waltham, MA, USA). Peptidylprolyl isomerase A (PPIA) mRNA was used to normalize RNA input. Relative quantification was performed in triplicate using 2^−ΔΔCT^.

**Western blotting**

Cells at 80%–90% confluence were lysed using RIPA buffer (Sigma-Aldrich, St. Louis, Missouri, US). Proteins (30-40 μg) were fractionated by SDS-PAGE and transferred to PVDF membranes (Bio-Rad Laboratories, CA, USA). Membranes were probed with the following antibodies: rabbit anti-HIF2α (ab243861, Abcam, Cambridge, UK) at 1:250 dilution; rabbit anti-HIF1α (Novus Biologicals, Centennial, CO, USA, Cat# NB100-449, RRID:AB_10001045) at 1:500 dilution; mouse anti-pan-cytokeratin AE1/AE3 (Santa Cruz Biotechnology (Dallas, TX, USA) Cat# sc-81714, RRID:AB_2191222) at 1:250 dilution; mouse anti-N-cadherin (Abcam Cat# ab19348, RRID:AB_444868) at 1:500 dilution; rabbit anti-vimentin (Abcam Cat# ab16700, RRID:AB_443435) at 1:500 dilution; rabbit anti-ZEB1 (Novus Cat# NBP1-77178, RRID:AB_11028448) at 1:500 dilution; rabbit anti-ZEB2 (Novus Cat# NBP1-82991, RRID:AB_11034164) at 1:500 dilution; rabbit anti-Snail2 (Cell Signaling Technology Danvers, MA, USA, Cat# 9585, RRID:AB_2239535) at 1:1,000 dilution; rabbit anti-phospho-mTOR (Ser2448) (Cell Signaling Technology Cat# 2971, RRID:AB_330970) at 1:1,000 dilution; rabbit anti-mTOR (7C10) (Cell Signaling Technology Cat# 2983, RRID:AB_2105622) at 1:1,000 dilution; rabbit anti-phospho-p44/42 MAPK (Erk1/2) (Thr202/Tyr204) (Cell Signaling Technology Cat# 9101, RRID:AB_331646) at 1:1,000 dilution; rabbit anti-p44/42 MAPK (Erk1/2) (Cell Signaling Technology Cat# 9102, RRID:AB_330744) at 1:1,000 dilution; rabbit anti-phospho-GSK-3β (Ser9) (Cell Signaling Technology Cat# 9336, RRID:AB_331405) at 1:1,000 dilution; rabbit anti-phospho-AKT (Ser473) (Cell Signaling Technology Cat# 9271, RRID:AB_329825) at 1:1,000 dilution; rabbit anti-pan-AKT (Abcam Cat# ab8805, RRID:AB_306791) at 1:500 dilution; rabbit anti-phospho-S6 ribosomal protein (Ser240/244) (D68F8) (Cell Signaling Technology Cat# 5364, RRID:AB_10694233) at 1:1,000 dilution; rabbit anti-S6 ribosomal protein (5G10) (Cell Signaling Technology Cat# 2217, RRID:AB_331355) at 1:1,000 dilution; rabbit anti-phospho-4E-BP1 (Thr37/46) (236B4) (Cell Signaling Technology Cat# 2855, RRID:AB_560835) at 1:1,000 dilution or rabbit anti- 4E-BP1 (53H11) (Cell Signaling Technology Cat# 9644, RRID:AB_2097841) at 1:1,000 dilution; rabbit anti-FAK (Abcam Cat# ab40794, RRID:AB_732300) at 1:500 dilution; rabbit anti-FAK, rabbit phospho-FAK (Tyr397) (Abcam Cat# ab81298, RRID:AB_1640500) at 1:500 dilution; mouse anti-c-Src (B-12) (Santa Cruz Biotechnology Cat# sc-8056, RRID:AB_627306) at 1:500 dilution; rabbit anti-phospho Src (Y419) (R and D Systems Cat# AF2685, RRID:AB_442167) at 1:200 dilution. Anti-β-actin (Sigma-Aldrich (St. Louis, MO, USA) Cat# A1978, RRID:AB_476692) was used as loading control. Bound antibodies were detected with IRDye 800 (Thermo Fisher Scientific Cat# SA5-10172, RRID:AB_2556752) or IRDye 680 IgG (LI-COR Biosciences, Lincoln, NE, USA, Cat# 926-32221, RRID:AB_621841) secondary antibodies (1:10,000, LI-COR Bioscience, Lincoln, NE, USA) and visualized using the Odyssey Fc Imaging System (LI-COR Biosciences, Lincoln, NE, USA).

**Lipid metabolism analysis**

Intracellular acetyl-CoA levels were measured with the Acetyl-Coenzyme A fluorometric assay kit (Sigma-Aldrich) following the manufacturer’s instructions. For lipid droplets analysis, Oil Red O (ORO) or BODIPY 493/503 (Thermo Fisher) staining were used. For ORO staining, cells at 100% confluence for 3 days were fixed with 10% formaldehyde for 1 hour, rinsed with 60% isopropanol for 5 minutes, stained with 3 mg/mL Oil Red O (ORO) for 10 min, and washed with water three times. In drug treatment experiments, cells were exposed to drugs for 72 hours. For BODIPY 493/503 staining, cells at 70-80% confluence were incubated with 10 μM BODIPY 493/503 for 30 min at 37 °C and visualized with a Zeiss Axio Observer (RRID:SCR_021351) microscope (Carl Zeiss, Germany). The ORO or BODIPY stained area were quantified using ImageJ software.

**Seahorse assay**

Cells were seeded in XFp cell culture miniplates (Agilent Technologies, Santa Clara, CA, USA) at a density of 1.2 x 10^4^ cells/well in 786-O or 1 x 10^4^ cells/well in RCC4. Twenty-four hours later, samples were analyzed to measure Oxygen Consumption Rate OCR with the Cell Mito Stress Test using the Agilent Seahorse XF HS Analyzer (RRID:SCR_019540) (Agilent Technologies) following manufacturer’s instructions. Concentration of inhibitors was as follows: Oligomycin (1.5 μM), Carbonyl Cyanite-4 (trifluoromethoxy) Phenylhydrazone (FCCP) (1 μM in 786-O and 2 μM in RCC4), and Rotenone/Antimycin A (R/AA) (0.5 μM). Data were normalized with the cell number following DAPI staining. Data analysis was performed using Agilent Seahorse Analytics (Agilent Technologies).

**Supplementary Figure legends**

**Supplementary Figure S1. DNA methylation across the *cPCDH* genes in ccRCC included in the TCGA database.** (A) Methylation levels at CpG sites within the promoter regions of the indicated protocadherins. Median values were calculated from 324 ccRCCs (red line) and 160 non-tumoral samples (blue line). Significantly hypermethylated CpG sites are highlighted by yellow circles. Data were analyzed with the Wanderer web tool. (B) Table shows methylation levels in *g*ene promoter regions in tumor tissues and non-tumoral counterparts. (C) Correlations between methylation levels and expression of the indicated *PCDHG* genes. Pearson coefficient (r) is shown.

**Supplementary Figure S2. Immunostaining of β-catenin in CT and C3KD cells**. Representative immunofluorescence images of β-catenin in the indicated 786-O cell lines showing its absence in the nuclei of both CT and C3KD cells.

**Supplementary Figure S3. Src expression and phosphorylation in control and *PCDHGC3*-knockdown cells**. Representative western blot showing total Src and phosphorylated Src (Y419) (p-Src) in control (CT) and *PCDHGC3*-knockdown (C3KD) ccRCC cells. β-actin was used as a loading control.

**Supplementary Figure S4. Analysis of the mTOR pathway and HIF2α expression in tumor xenografts.** (A-C) Representative images of western blots and the respective quantifications of the indicated proteins involved in the mTOR and ERK pathways (A, B) and the HIF2α protein (C) in three independent CT and C3KD derived tumor xenografts (D) Representative immunohistochemical images of HIF2α in CT and C3KD tumor xenograft. Scale bars = 200 μm. *p < 0.05, **p < 0.01.

**Supplementary Figure S5. mTOR and HIF2α inhibitors suppress high proliferative rate induced by *PCDHGC3* deficiency in RCC4 cells.** (A) Real-time analysis of cell proliferation with the iCELLigence system. (B, C) Colony formation assays in RCC4 CT (B) and C3KD (C) cells after treatment with the indicated drugs. (D) Representative fluorescence XYZ projections of CT or C3KD RCC4 printed cells treated with the indicated drugs. *p < 0.05, *** p < 0.001.

**Supplementary Figure S6. Analysis of mTOR pathway (A) and HIF2α** **(B) expression in tumoral and metastatic tissues raised in the orthotopic xenograft model.** Representatives immunoblot images and quantifications of the indicated proteins in independent primary tumors and metastasis (Mt). *p < 0.05.

**Supplementary Figure S7. Analysis of mitochondrial function in CT and C3KD cells**. Seahorse Cell Mito Stress Test assays performed in CT and C3KD 786-O and RCC4 cells. Data are represented as oxygen consumption rate (OCR). Arrows indicate additions of the indicated stressors: oligomycin (Olig), carbonyl cyanite-4 (trifluoromethoxy) phenylhydrazone (FCCP) and rotenone/antimycin A (R/AA).

**Supplementary Figure S8. Differential effects of temsirolimus and PT2385 on lipid droplet accumulation**

Representative images of phase-contrast microscopy of the indicated cells stained with Oil Red O (ORO) three days after reaching confluence. Cells were treated with temsirolimus (1 µM) and/or PT2385 (50 µM) for 72 hours post-confluence. Temsirolimus reduced lipid droplet accumulation in both CT and C3KD cells, whereas PT2385 specifically affected lipid droplets in C3KD cells. Quantification of lipid droplet area is presented in Figure 7H.

**Supplementary Table S1. Proteins differentially expressed in CT versus C3KD 786-O cells.**

**Supplementary Table S2. Detailed data on GO terms and pathways enriched in CT versus C3KD 786-O cells based on proteomic analysis.**
