# Supplementary figures and images for: PCDHGC3 silencing promotes clear cell renal cell carcinoma metastasis via mTOR/HIF2α activation, lipid metabolism rewiring, and ferroptosis evasion
Source: Cell Death Dis. 2026 Mar 26;17(1):409. doi: 10.1038/s41419-026-08643-y (PMC13144475; doi:10.1038/s41419-026-08643-y)

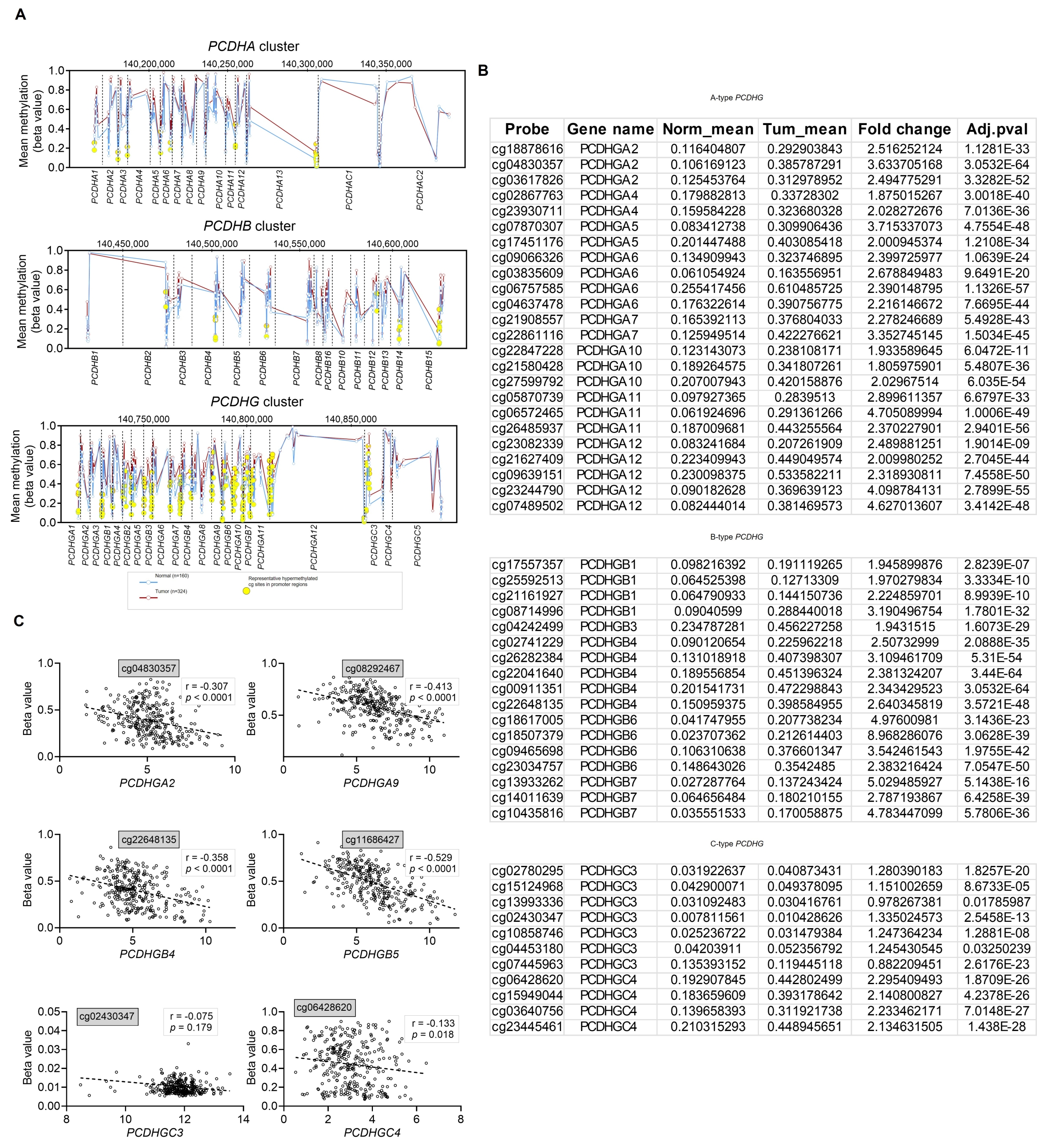

Supplement: Supplementary file 2 — Supplementary Figure S1 [file 41419_2026_8643_MOESM2_ESM.jpg]

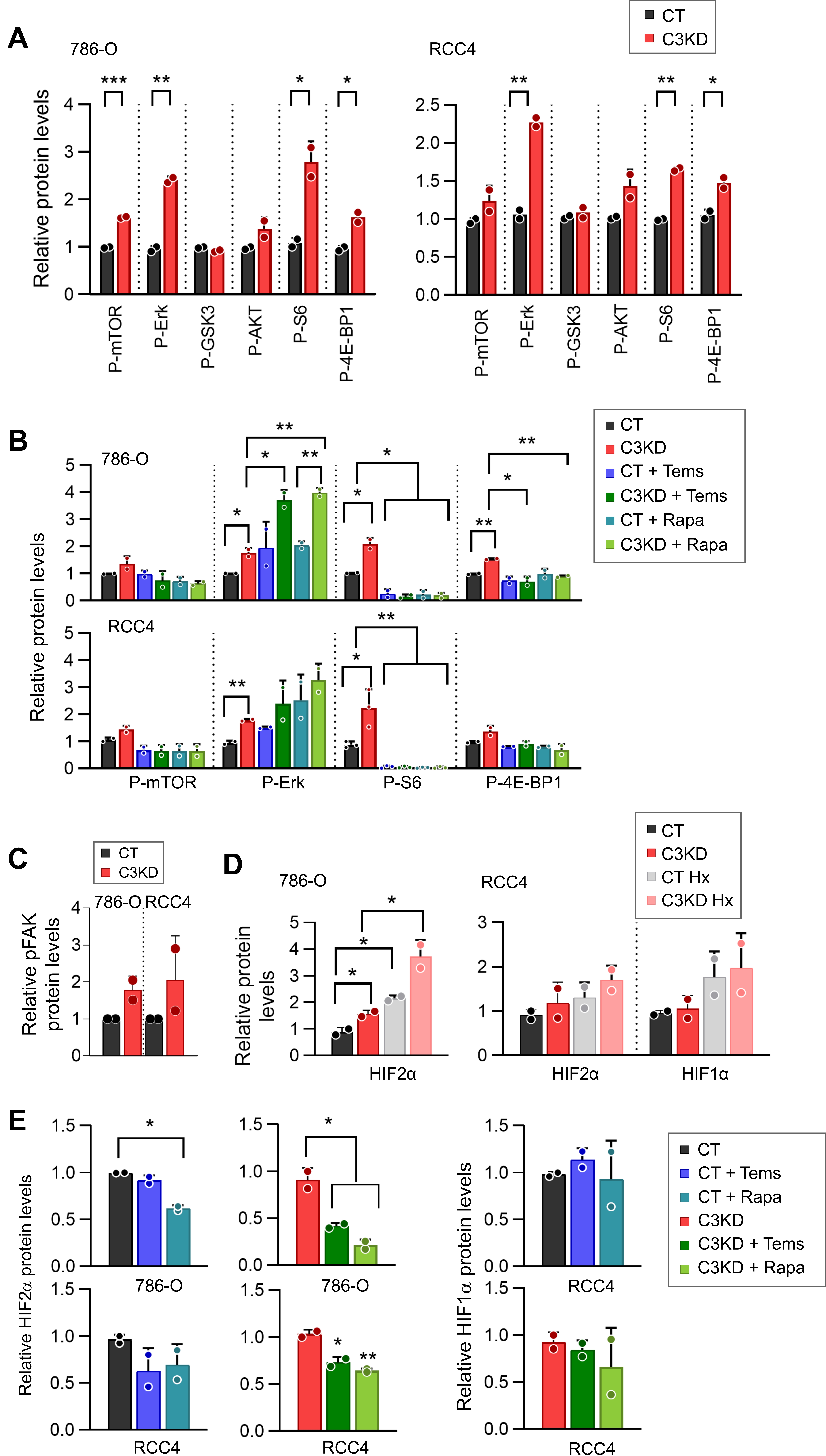

Supplement: Supplementary file 3 — Supplementary Figure S2 [file 41419_2026_8643_MOESM3_ESM.jpg]

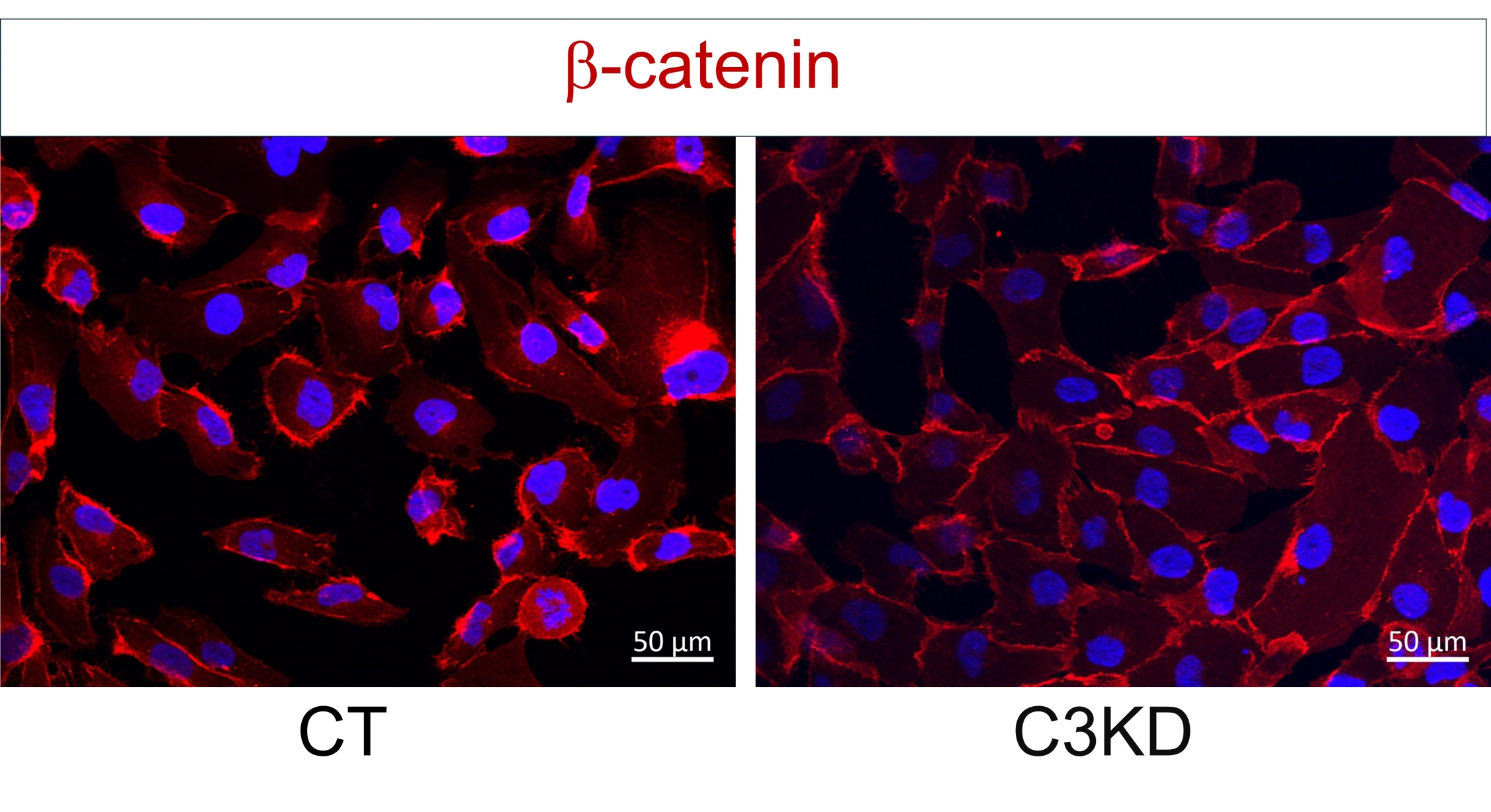

Supplement: Supplementary file 4 — Supplementary Figure S3 [file 41419_2026_8643_MOESM4_ESM.jpg]

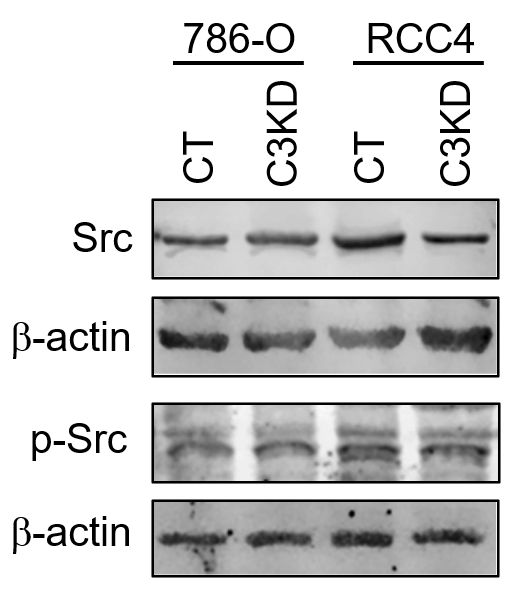

Supplement: Supplementary file 5 — Supplementary Figure S4 [file 41419_2026_8643_MOESM5_ESM.jpg]

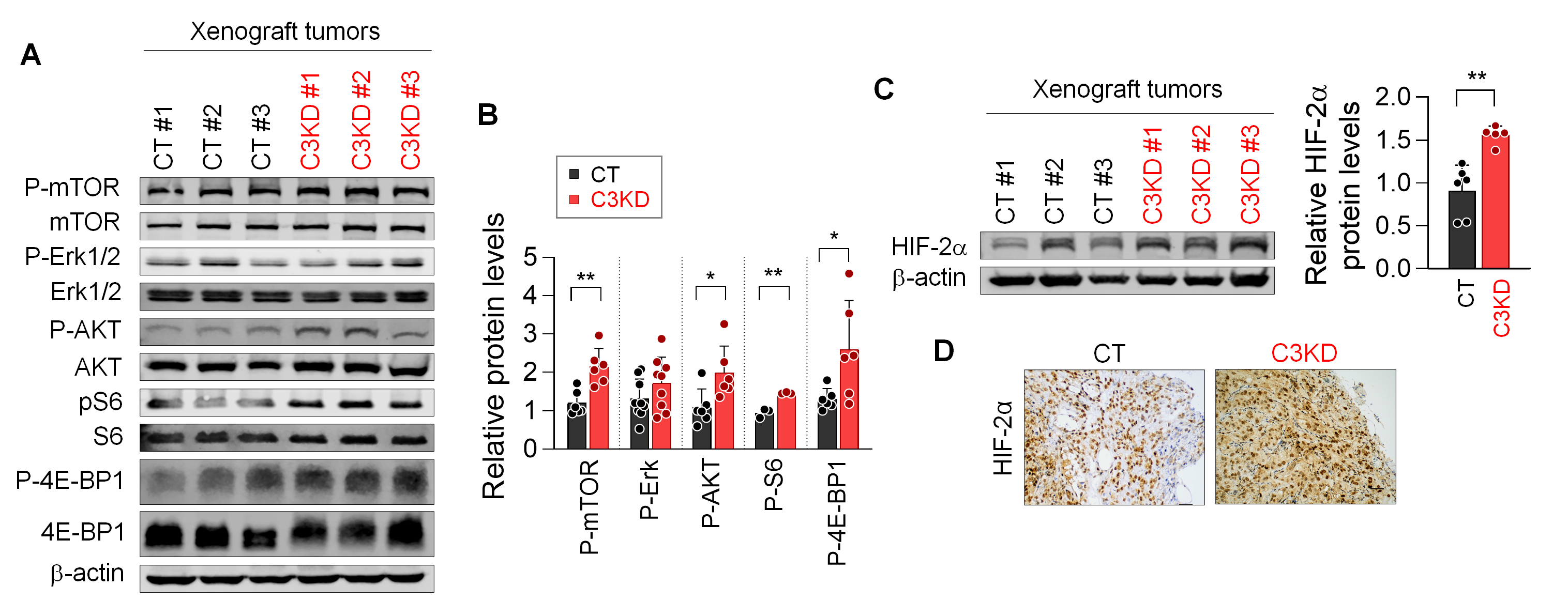

Supplement: Supplementary file 6 — Supplementary Figure S5 [file 41419_2026_8643_MOESM6_ESM.tif]

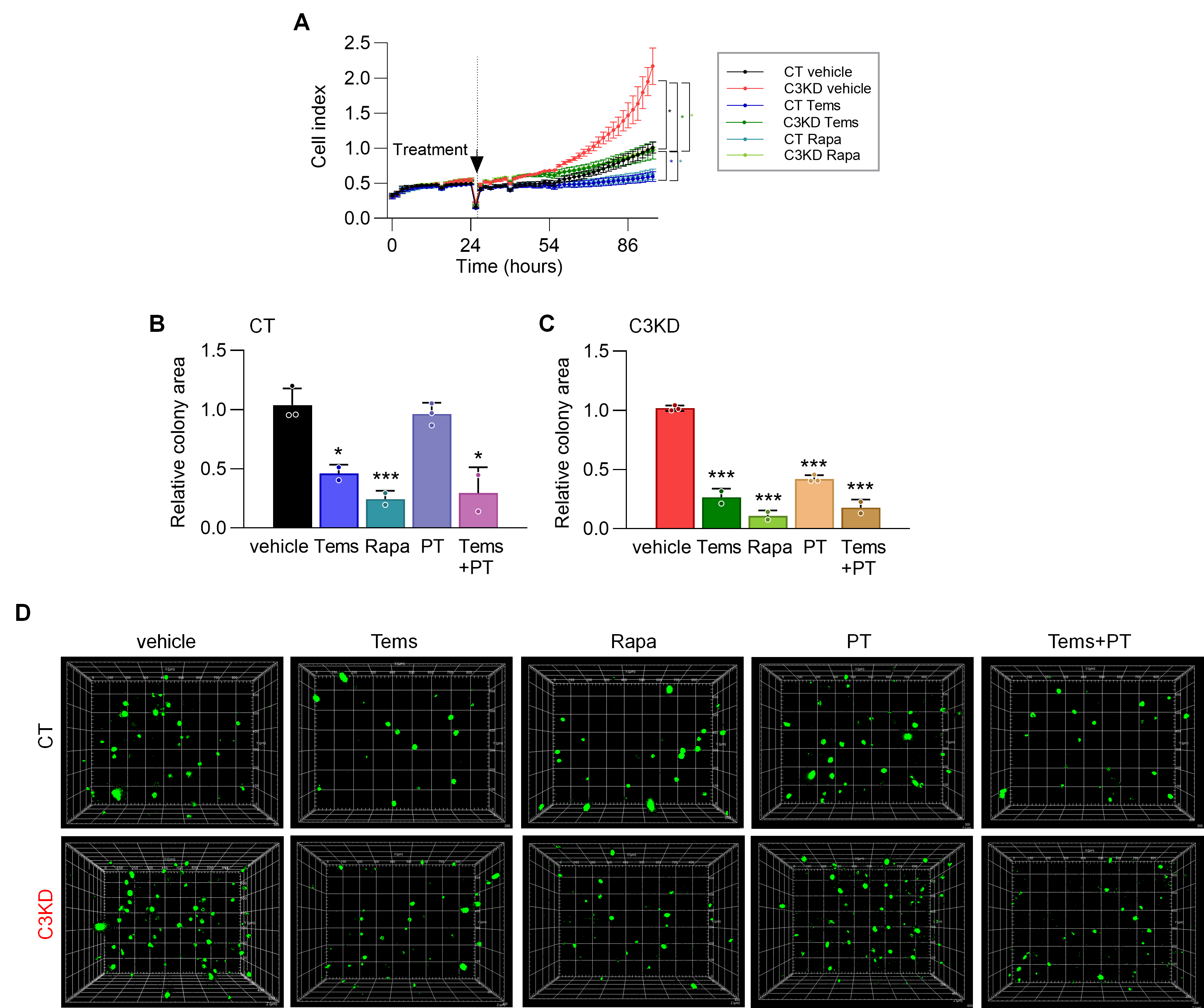

Supplement: Supplementary file 7 — Supplementary Figure S6 [file 41419_2026_8643_MOESM7_ESM.tif]

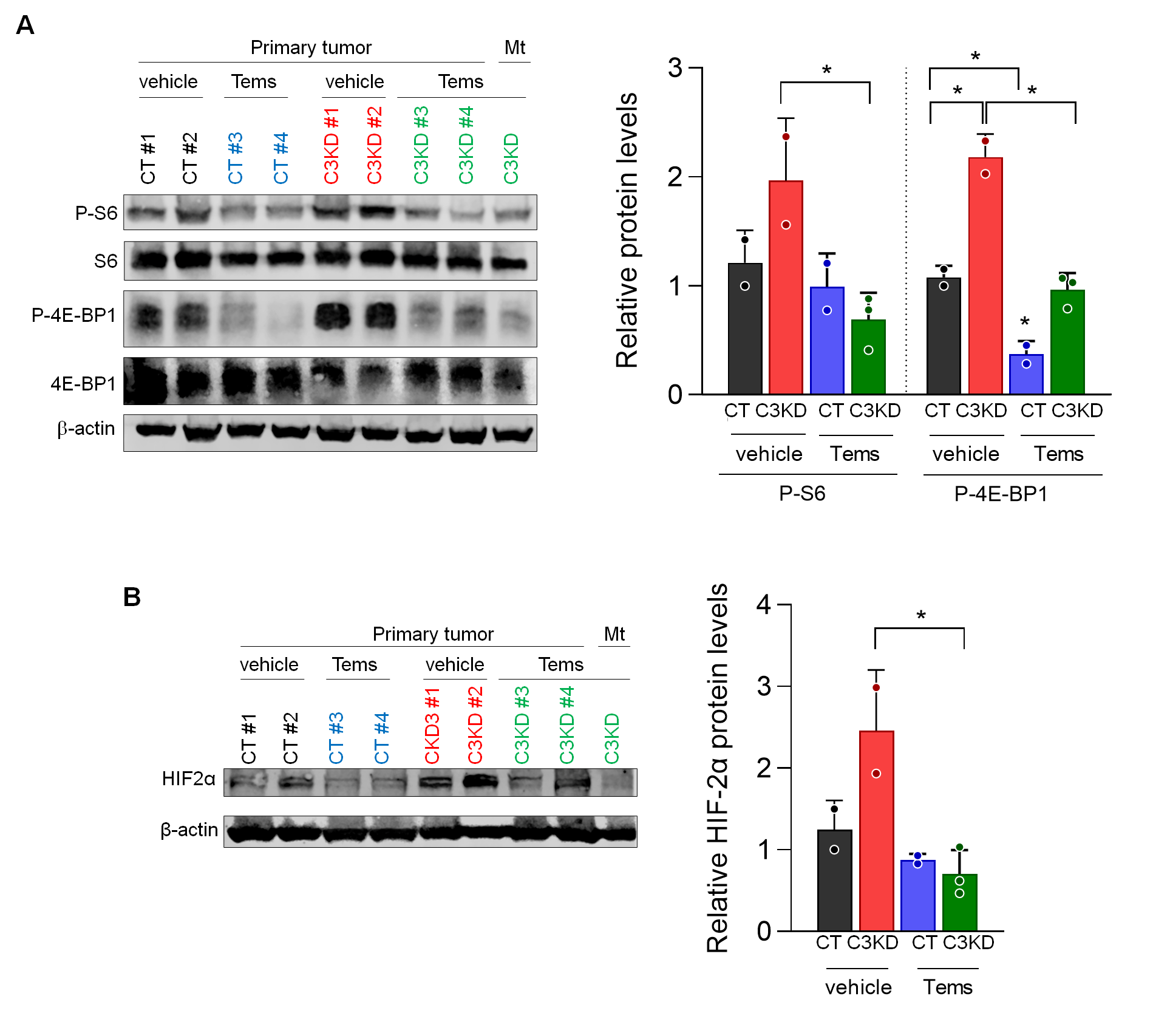

Supplement: Supplementary file 8 — Supplementary Figure S7 [file 41419_2026_8643_MOESM8_ESM.tif]

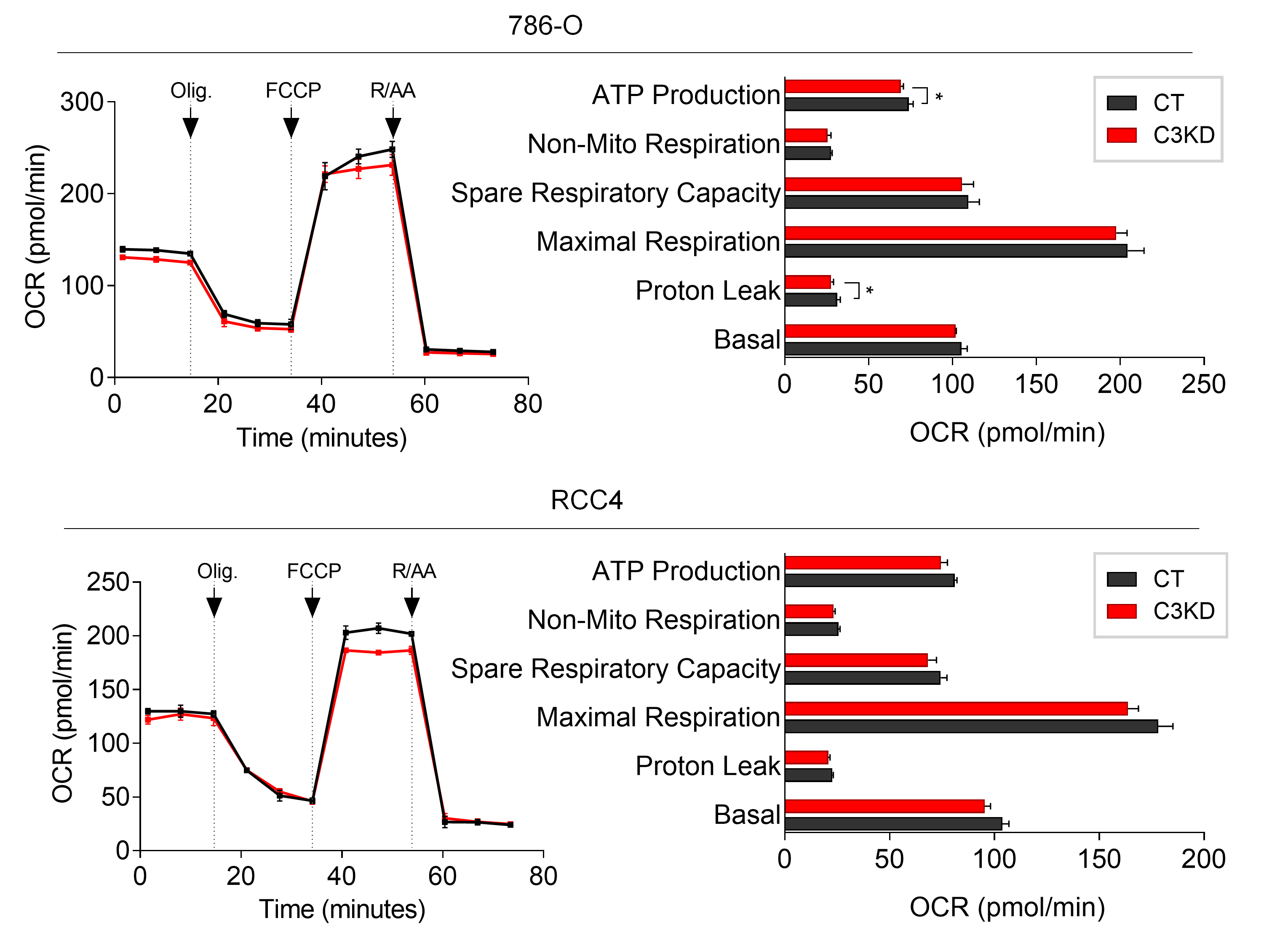

Supplement: Supplementary file 9 — Supplementary Figure S8 [file 41419_2026_8643_MOESM9_ESM.tif]

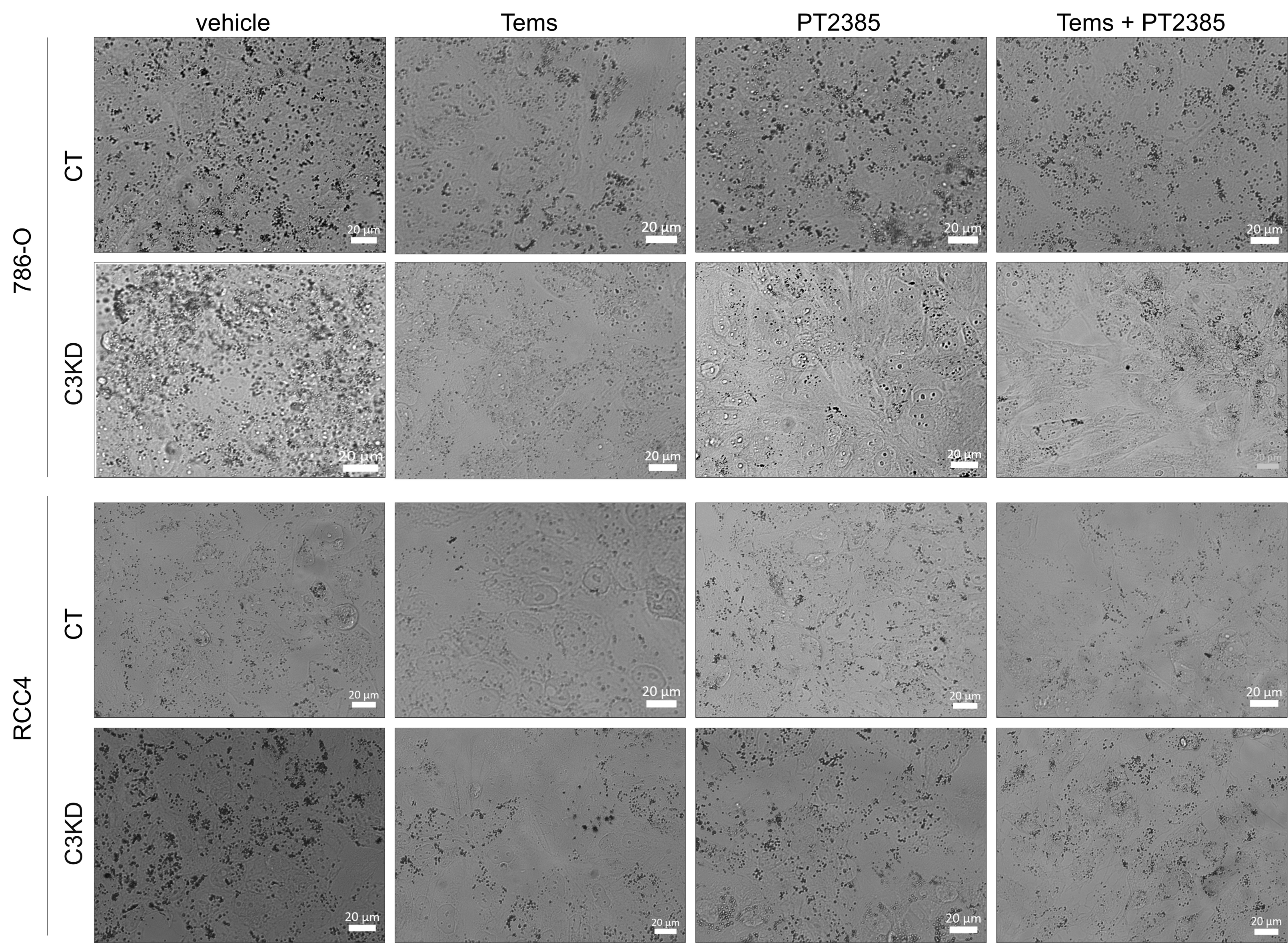

Supplement: Supplementary file 10 — Supplementary Figure S9 [file 41419_2026_8643_MOESM10_ESM.jpg]
