# Supplementary material for: PCDHGC3 silencing promotes clear cell renal cell carcinoma metastasis via mTOR/HIF2α activation, lipid metabolism rewiring, and ferroptosis evasion
Source: Cell Death Dis. 2026 Mar 26;17(1):409. doi: 10.1038/s41419-026-08643-y (PMC13144475; doi:10.1038/s41419-026-08643-y)
Supplement: Supplementary file 12 — Table S2 [file 41419_2026_8643_MOESM12_ESM.docx]

**Upregulated MSigDB_Hallmark**

**Upregulated Reactome**
