# Supplementary material for: PCDHGC3 silencing promotes clear cell renal cell carcinoma metastasis via mTOR/HIF2α activation, lipid metabolism rewiring, and ferroptosis evasion
Source: Cell Death Dis. 2026 Mar 26;17(1):409. doi: 10.1038/s41419-026-08643-y (PMC13144475; doi:10.1038/s41419-026-08643-y)

**Figure 3B**

Pan-CK

CT

C3KD

β-actin

N-cadh

CT

C3KD

β-actin

ZEB1

CT

C3KD

786-O

ZEB2

CT

C3KD

CT

C3KD

786-O

RCC4

β-actin

β-actin

Vimentin

β-actin

CT

C3KD

RCC4

ZEB1

β-actin

CT

C3KD

RCC4

Pan-CK

β-actin

CT

C3KD

CT

C3KD

N-cadh

RCC4

786-O

β-actin

Snail2

β-actin

CT

C3KD

CT

C3KD

786-O

RCC4

RCC4

786-O

CT

C3KD

CT

C3KD

786-O

RCC4

**Figure 4A**
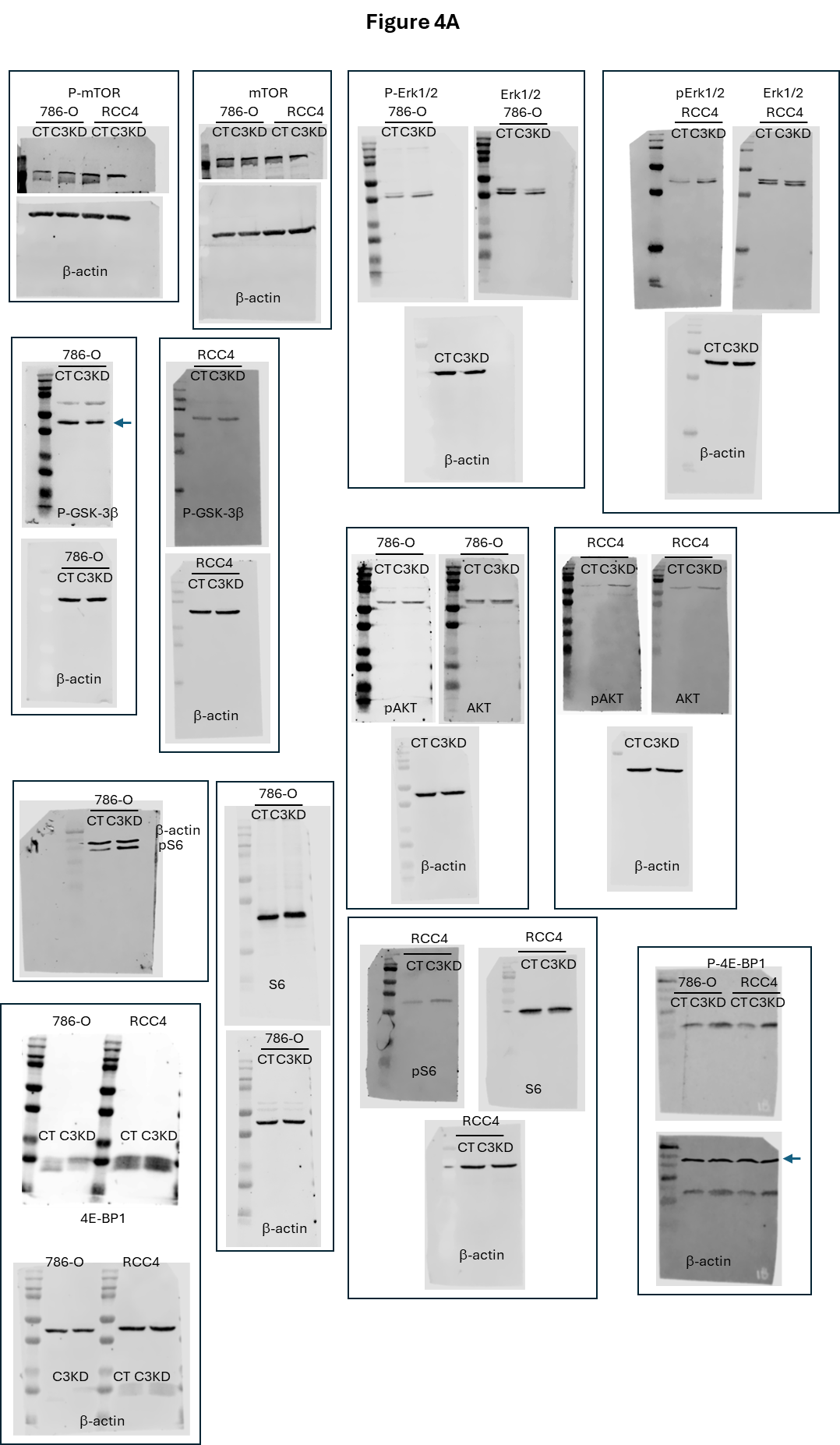


**Figure 4B**

**
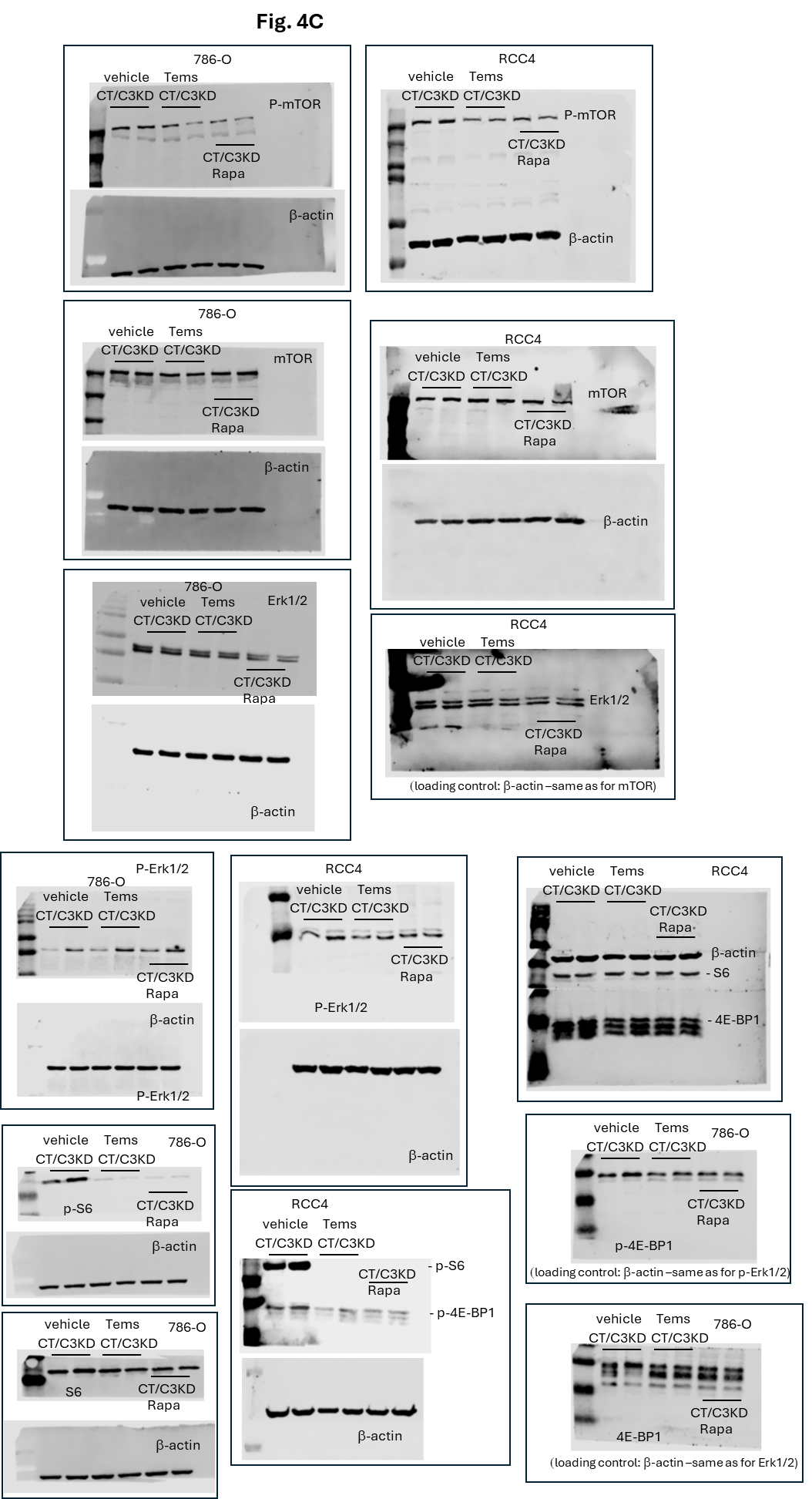
**

**Figure 4D**

p-FAK (phospho Y397)

CT

C3KD

RCC4

CT

C3KD

786-O

β-actin


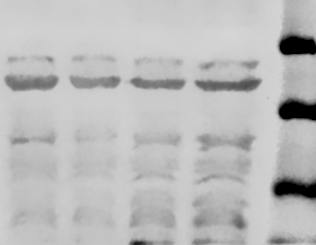


FAK

β-actin

CT

C3KD

RCC4

CT

C3KD

786-O

**Figure 4E**

**
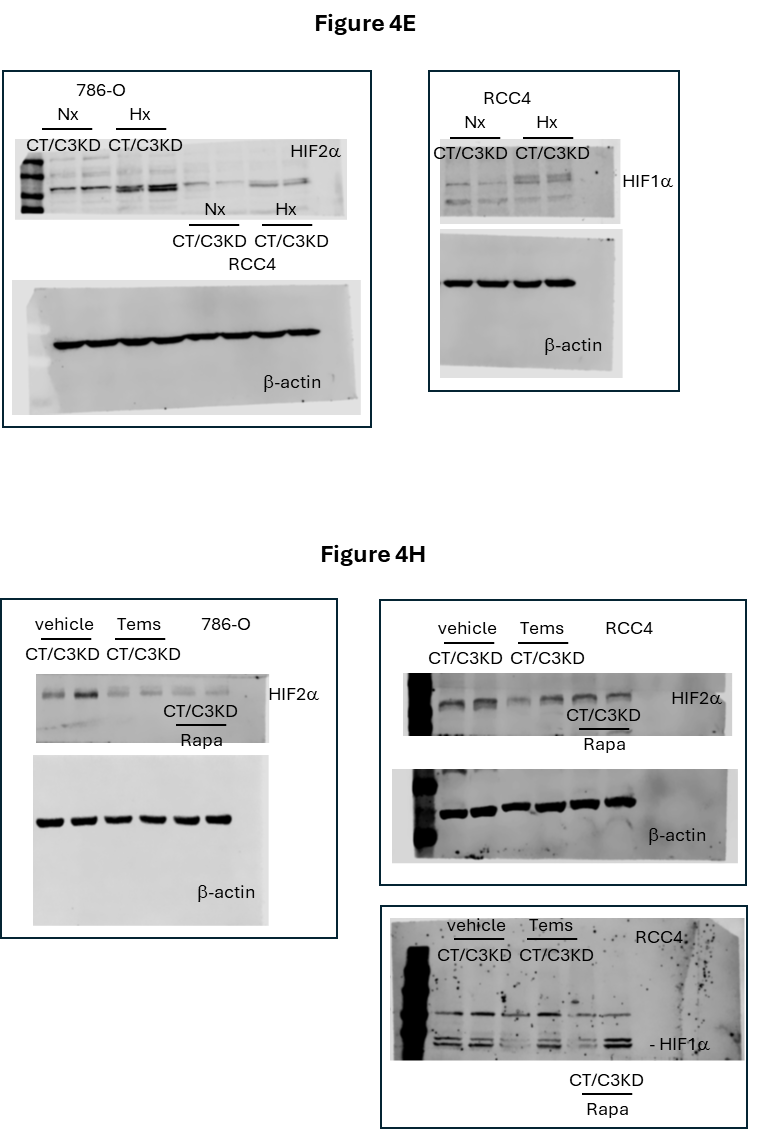
**

**Figure 4G**

**
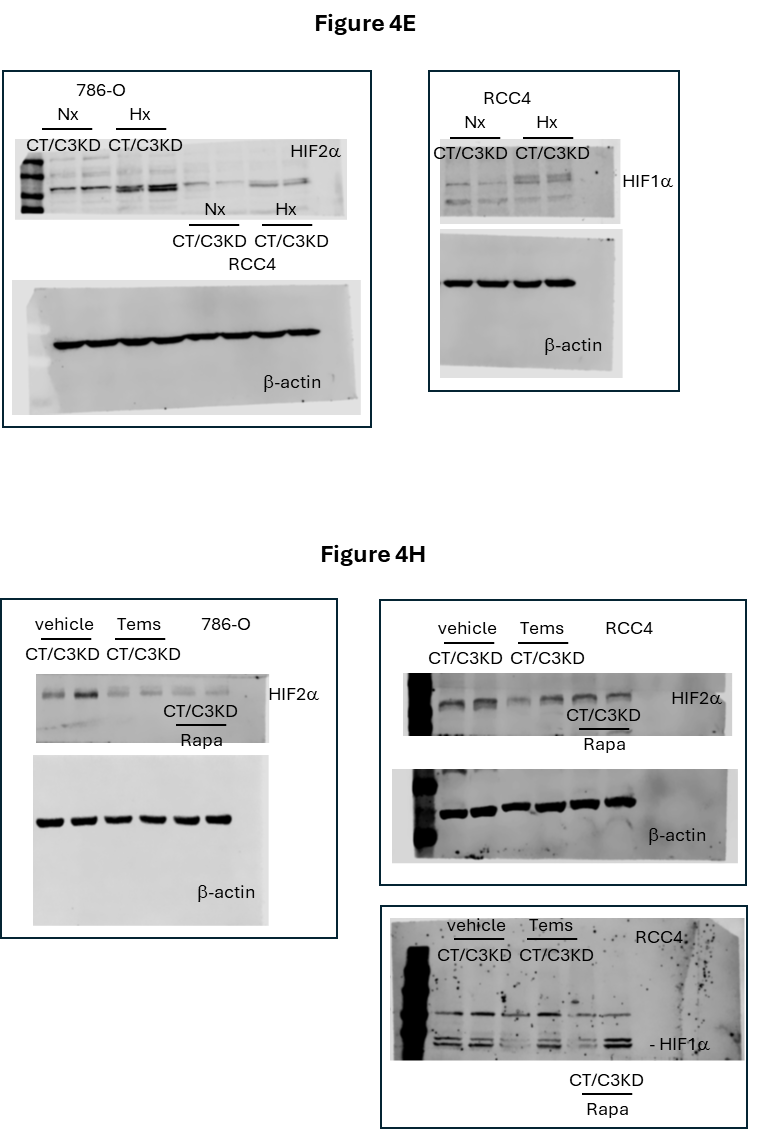
**

**Supplementary Fig. S4**

**
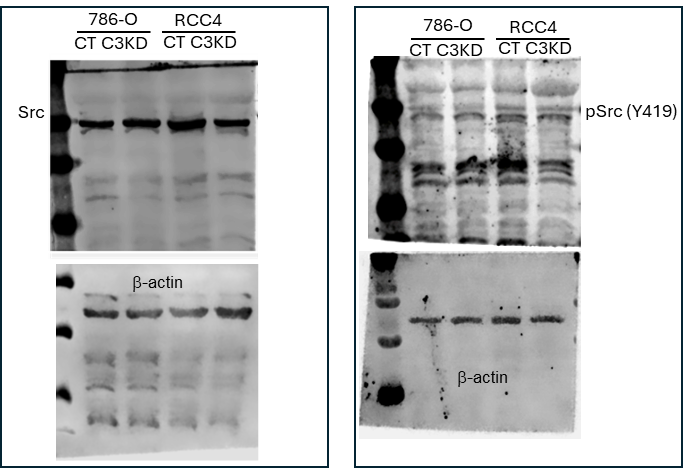
**

**Supplementary Fig. S5A**

**
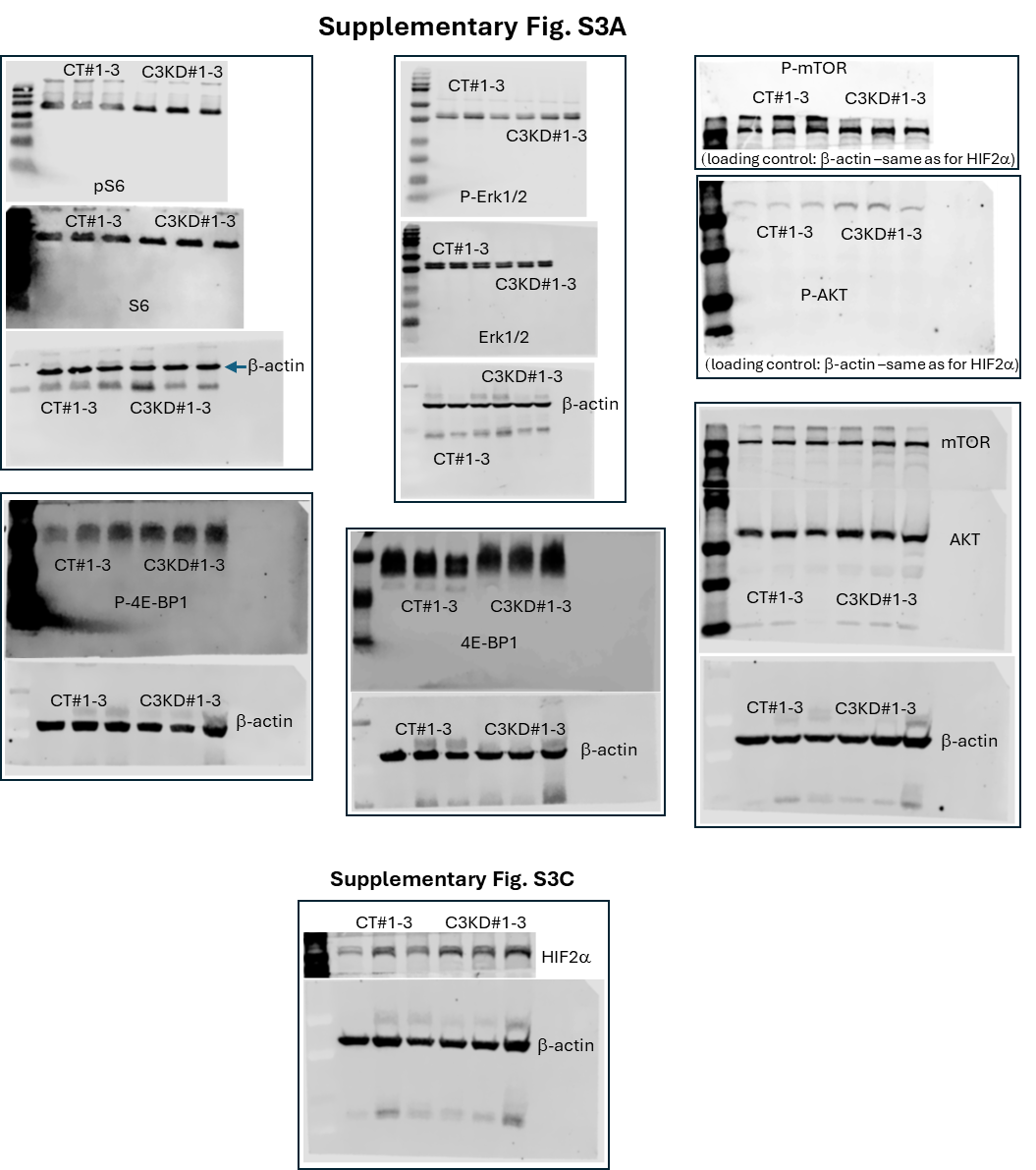
**

**
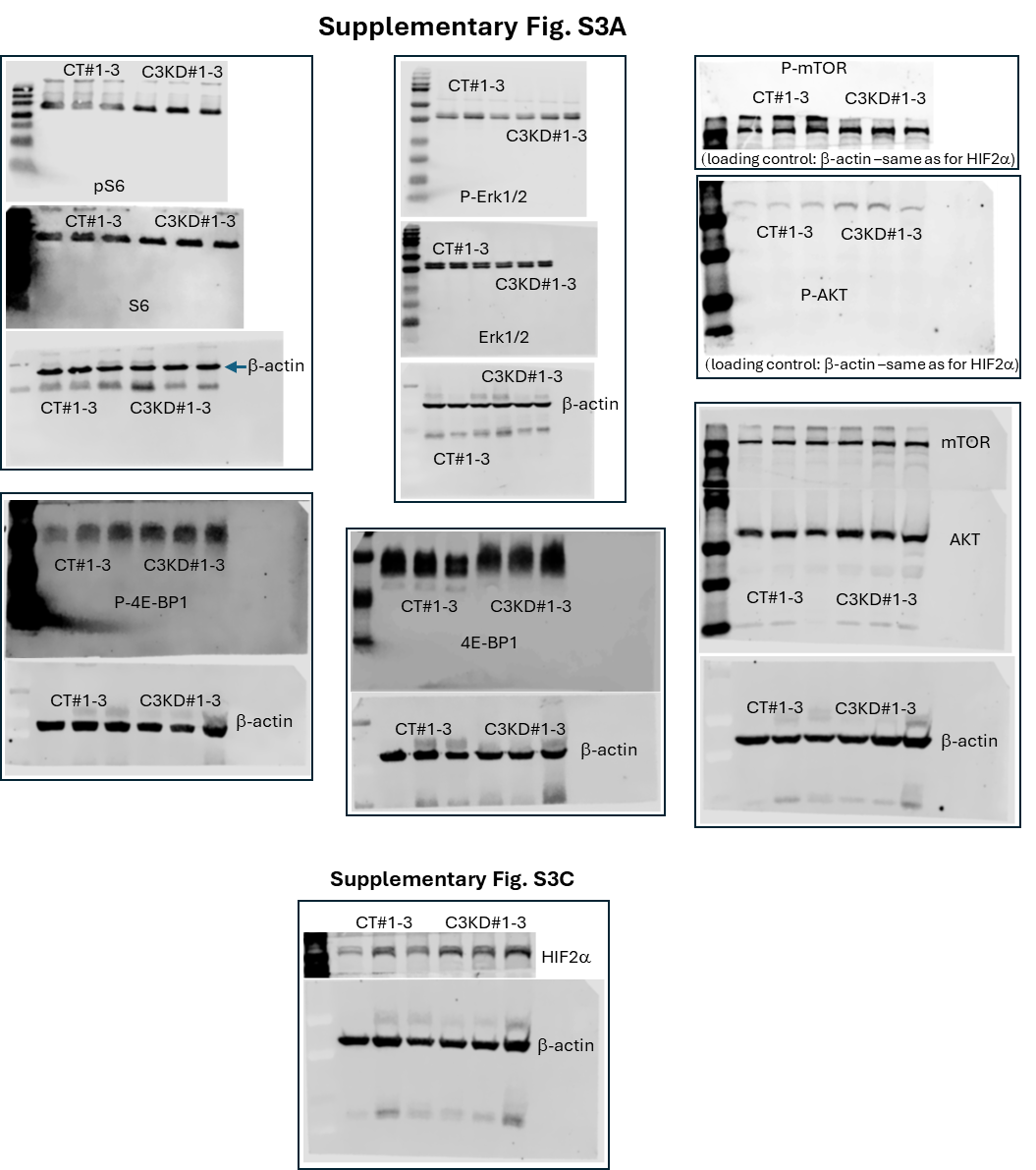
Supplementary Fig. S5C**

**Supplementary Figure S7**


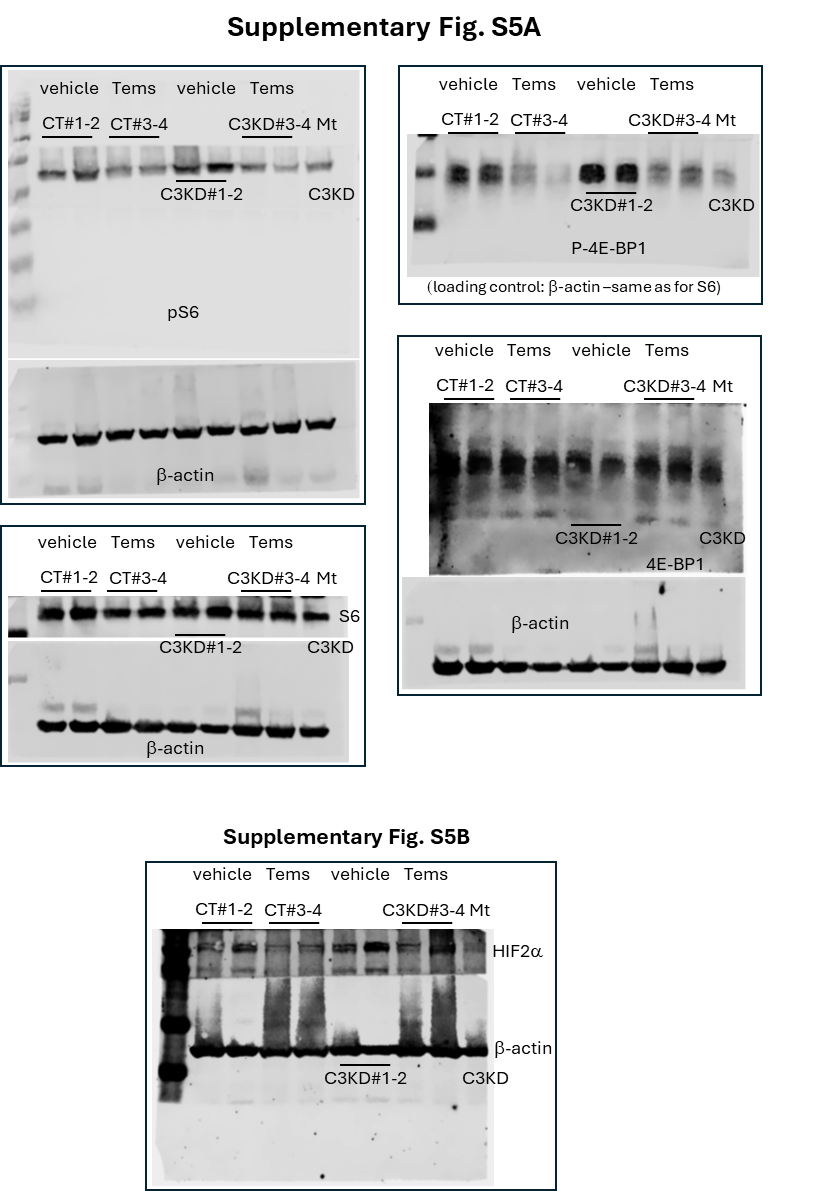

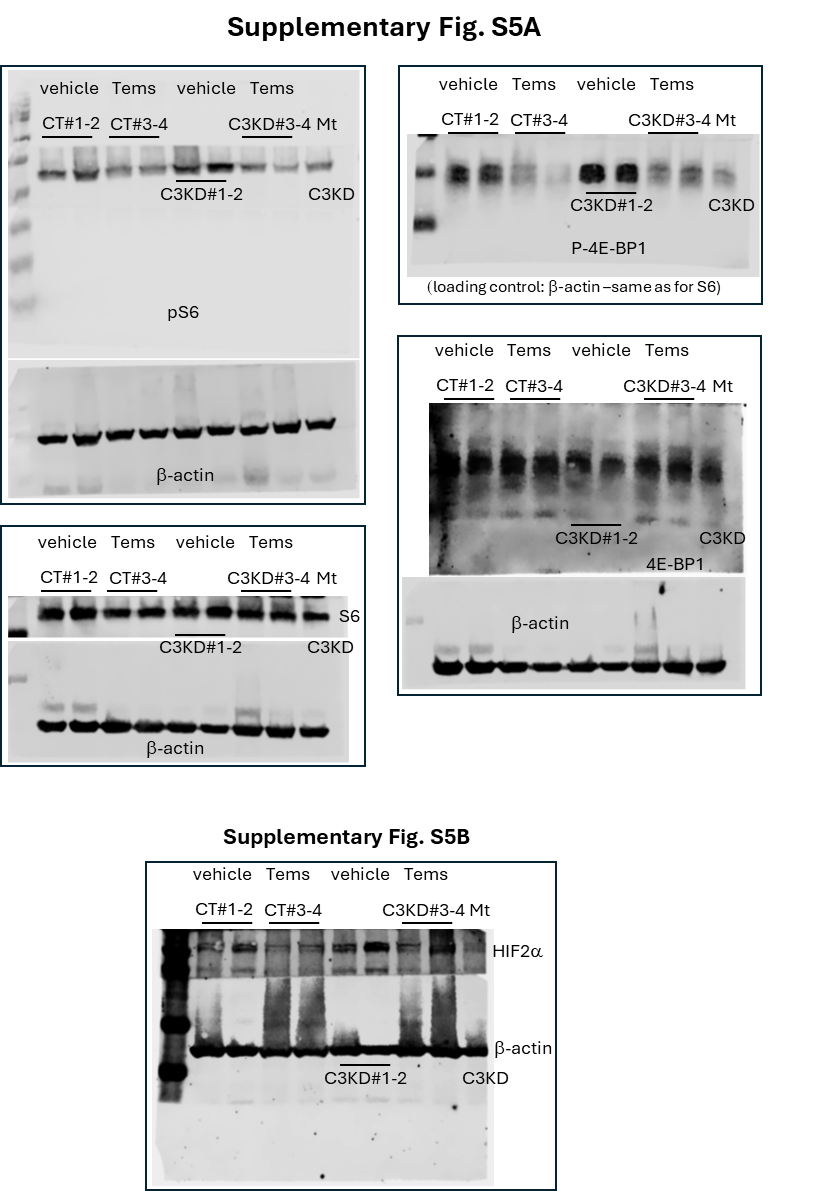

Supplement: Supplementary file 13 — Uncropped western blots [file 41419_2026_8643_MOESM13_ESM.docx]
